# Ruthenium(II)/(III) DMSO-Based Complexes of 2-Aminophenyl Benzimidazole with In Vitro and In Vivo Anticancer Activity

**DOI:** 10.3390/molecules25184284

**Published:** 2020-09-18

**Authors:** Shadia A. Elsayed, Shane Harrypersad, Heba A. Sahyon, Mohammed Abu El-Magd, Charles J. Walsby

**Affiliations:** 1 Chemistry Department, Faculty of Science, Damietta University, New Damietta 34517, Egypt; 2 Department of Chemistry, Simon Fraser University, 8888 University Dr, Burnaby, BC V5A 1S6, Canada; shane_harrypersad@sfu.ca; 3 Chemistry Department, Faculty of Science, Kafrelsheikh University, Kafrelsheikh 33516, Egypt; 4 Anatomy and Embryology Department, Faculty of Veterinary Medicine, Kafrelsheikh University, Kafrelsheikh 33516, Egypt; mohrizk73@yahoo.com

**Keywords:** ruthenium, benzimidazole, anticancer, DNA, in vitro cytotoxicity, in vivo activity

## Abstract

New anticancer ruthenium(II/III) complexes [RuCl_2_(DMSO)_2_(Hapbim)] (**1**) and [RuCl_3_(DMSO) (Hapbim)] (**2**) (Hapbim = 2-aminophenyl benzimidazole) have been synthesized and characterized, and their chemotherapeutic potential evaluated. The interaction of the compounds with DNA was studied by both UV-Visible and fluorescence spectroscopies, revealing intercalation of both the Hapbim ligand and the Ru complexes. The in vitro cytotoxicity of the compounds was tested on human breast cancer (MCF7), human colorectal cancer (Caco2), and normal human liver cell lines (THLE-2), with compound (**2**) the most potent against cancer cells. The cytotoxic effect of (**2**) is shown to correlate with the ability of the Ru(III) complex to induce apoptosis and to cause cell-cycle arrest in the G2/M phase. Notably, both compounds were inactive in the noncancerous cell line. The anticancer effect of (**2**) has also been studied in an EAC (Ehrlich Ascites Carcinoma) mouse model. Significantly, the activity of the complex was more pronounced in vivo, with removal of the cancer burden at doses that resulted in only low levels of hepatotoxicity and nephrotoxicity. An apoptosis mechanism was determined by the observation of increased *Bax* and caspase *3* and decreased *Bcl2* expression. Furthermore, (**2**) decreased oxidative stress and increased the levels of antioxidant enzymes, especially SOD, suggesting the enhancement of normal cell repair. Overall, compound (**2**) shows great potential as a chemotherapeutic candidate, with promising activity and low levels of side effects.

## 1. Introduction

Although cisplatin has been clinically approved as an anticancer agent against several types of cancers, its use can be limited by severe side effects, such as nephrotoxicity and hepatotoxicity, in addition to development of resistance to the drug [1]. For these reasons, interest in the development of other non-platinum-based drugs with lower toxicity and reduced susceptibility to drug resistance has increased significantly [2,3,4,5,6]. Ruthenium-based anticancer drugs are particularly promising as next-generation metal-based chemotherapeutics [7,8,9,10]. Ru(II) and Ru(III) complexes have several advantages over platinum anticancer compounds, including: (i) Octahedral geometry, which allows for structure diversity (as compared to the square-planar geometry in cisplatin), (ii) a range of oxidation states that are accessible under physiological conditions (+2 to +4), and (iii) they often exhibit slow ligand-exchange kinetics [6,11,12,13]. The Ru(III) complexes NAMI-A, ImH [*trans*-Ru(III)Cl_4_(DMSO-S)Im] (Im = imidazole) and KP1019, IndH *trans*-[Ru(III)Cl_4_(Ind)_2_] (Ind = Indazole) and sodium *trans*-[Ru(III)Cl_4_(Ind)_2_] (NKP-1339) have all successfully reached human clinical trials [13,14,15], which has stimulated development of related ruthenium complexes, including those with DMSO ligands [16,17,18,19]. Both NAMI-A and KP1019/NKP-1339 have properties that are distinct from common platinum anticancer drugs. NAMI-A is active against tumor metastases [13], whereas KP1019 and NKP-1339 directly cause apoptotic cell death through the intrinsic mitochondrial pathway and the formation of reactive oxygen species [17,20]. Furthermore, the cytotoxicity of many ruthenium(III) prodrugs has been linked to the reduction of Ru(III) to Ru(II) species in vivo [20,21], although the role this plays in the activity of the complexes remains controversial [22].

Ligand design as a key aspect in the development of new ruthenium complexes as anticancer agents. Heterocyclic nitrogen-containing ligands are interesting compounds in themselves due both to their structural diversity and variety of pharmaceutical applications. Benzimidazole-containing compounds are a class of these compounds that often exhibit anti-inflammatory, antifungal, antibacterial, antioxidant, and antitumor activity [23,24]. These compounds also readily coordinate to metal ions, and their structure, flexibility, and bulkiness can all affect the cytotoxic properties of the resulting complexes [25]. Consequently, a variety of different transition-metal complexes with benzimidazole ligands have been reported in medicinal applications [24,26,27]. For example, Ru(II), Rh(III), and Ir(III) complexes of 2-aminophenyl benzimidazole have strong binding affinities for both DNA and proteins and show promising cytotoxicity and in vitro anticancer activity against the SiHa human cervical cancer cell line [24,28]. Similarly, coordination of 2-aminoethyl benzimidazole to Ru(III) was reported to give a complex with in vitro and in vivo cytotoxic activity against human breast MCF7 and colon HCT116 cell lines [29].

**Figure 1:**
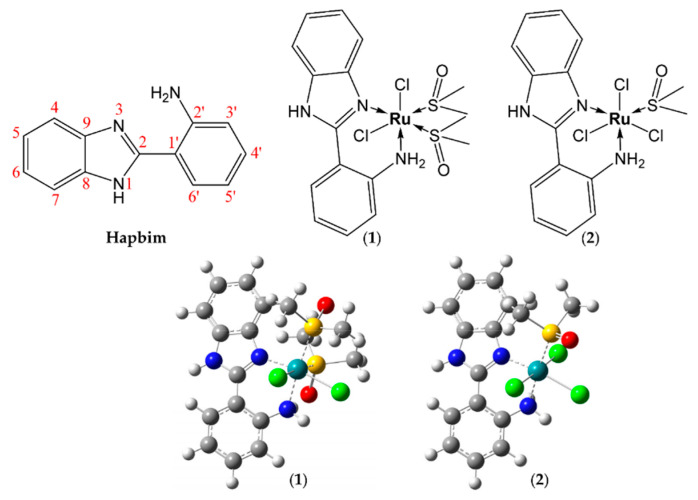
Chemical structures of 2-aminophenyl benzimidazole ligand (**Hapbim**), [Ru(II)Cl_2_(DMSO)_2_(Hapbim)] (**1**) and [Ru(III)Cl_3_(DMSO)(Hapbim)] (**2**), and DFT calculated structures of metal complexes.

It has been shown that ruthenium-based DMSO complexes, such as *cis*-RuCl_2_(DMSO)_4_, have a high affinity for N-donor ligands, potentially generating compounds with in vitro and in vivo anticancer activity [30]. In this work, motivated by the interesting anticancer properties of both benzimidazole and DMSO ruthenium complexes, we have synthesized two new ruthenium-DMSO-based complexes of 2-aminophenyl benzimidazole (Hapbim); [Ru(II)Cl_2_(DMSO)_2_(Hapbim)] (**1**) and [Ru(III)Cl_3_(Hapbim)(DMSO)] (**2**) (Figure 1). We report their redox properties, solution behavior, DNA binding, and in vitro cytotoxic properties against normal and cancerous cell lines. Furthermore, the in vivo anticancer activity against Ehrlich carcinoma (EAC) was also evaluated in a mouse model indicating that (**2**), in particular, is a promising chemotherapeutic candidate.

## 2. Results and Discussion

### 2.1. Synthesis and Characterization

#### 2.1.1. Synthesis

The complexes (**1**) and (**2**) were prepared by refluxing the ligand, Hapbim, with *cis*-[RuCl_2_(DMSO)_4_] or [DMSO)_2_H][*trans*-RuCl_4_(DMSO)_2_], respectively in a 1:1 molar ratio in ethanol for 2 h. Both complexes are colored, powder-like, and well soluble in organic solvents such as DMF and DMSO but less soluble in water, methanol and ethanol, dichloromethane, and acetonitrile. The data obtained from elemental analyses and spectroscopic measurements (IR, ^1^H, ^13^C NMR, HRESI-MS, UV-Vis) are in close agreement with the proposed structures of the complexes (Figure 1). The molar conductivity of the complexes was measured at room temperature in DMF (10^−3^ M), the values obtained (14 and 18 Ω^−1^cm^2^mol^−1^ respectively) indicate the non-electrolytic nature of the complexes in the DMF solution. Multiple attempts to grow crystals suitable for X-ray analysis, under a variety of conditions, were unsuccessful.

#### 2.1.2. FTIR Spectra

The FTIR spectrum of the Hapbim ligand shows characteristic bands at 3383 and 3140 cm^−1^ assigned to the stretching vibrations of NH_2_ and ν(NH) of benzimidazole, respectively [23,31,32,33]. In the complexes, the band due to NH_2_ was shifted to a lower frequency (3240–3251 cm^−1^) as a result of the coordination, while the NH band did not show a significant shift, confirming non-coordination to the metal center. A sharp band observed at 1608 cm^−1^ is assigned to ν(C=N) and was shifted to a higher frequency in the complexes (1620–1626 cm^−1^) due to the coordination of *N*-cyclic azomethine. Hence, the Hapbim ligand coordinates in a neutral bi-dentate fashion. In the FTIR of the precursor *cis*-[RuCl_2_(DMSO)_4_], the two sharp bands that appeared at 1097 and 1020 cm^−1^ are due to ν(SO) of the *S*-bonded DMSO, whereas *O*-bonded DMSO would be expected to appear at around 930 cm^−1^ [34]. In complex (**1**), the first two bands appeared at 1051 and 1015 cm^−1^, respectively, while the latter band disappeared, which confirms the replacement of an O-bonded DMSO and an S-bonded DMSO by the bi-dentate Hapbim ligand [35,36,37]. The observation of new bands near 442 cm^−1^ (for (**1**)) and 445 cm^−1^ (for (**2**)) are assigned to ν(Ru-N) stretching vibration [38]. Additionally, the water of hydration for complex (**2**) was observed as a broad band at 3470 cm^−1^. IR spectral data and assignments are listed in the Materials and Methods Section and Figure S1a–c, Supplementary Materials.

#### 2.1.3. NMR Spectra

The structure of the Hapbim ligand with its atom labeling scheme are presented in Figure 1. The ^1^H-NMR spectrum of Hapbim (Figure S2a,b) displays two singlets at δ 12.66 and 7.26 ppm due to NH and NH_2_ groups, respectively. In addition, four doublets at δ 6.83, 7.84, 7.65, and 7.52 ppm are assigned to H(3′), H(6′), H(7), and H(4), respectively, and the doublet of doublets and multiplets at 6.66 ppm and in the 7.14–7.23 ppm regions are attributed to H(4′), H(5′), and H(5,6) [23,24,33]. The ^1^H-NMR spectrum of the diamagnetic Ru(II) complex (**1**) (Figure S2c,d) showed a downfield shift of the NH signal (δ 13.53) and the NH_2_ protons become inequivalent and appeared separately at δ 6.18 (d, *J* = 11 Hz) and 7.15 (d, *J* = 11 Hz) ppm (as determined by ^1^H-NMR and ^1^H–^1^H COSY). Moreover, the resonances arising from other protons were also shifted downfield, which is likely due to the reduction of electron density resulting from the electron-withdrawing effect of the metal ion. The coordination of S-bonded DMSO molecules to the Ru(II) ion was confirmed by peaks observed at δ 3.02, 3.23, 3.34 (coincident with H_2_O signal), and 3.48 ppm. As expected, no peaks were observed for *O*-bonded DMSO, which is predicted to show a lower ^1^H signal at around 2.7 ppm [39]. The paramagnetic ^1^H-NMR for the Ru(III) complex (**2**) (Figure S2e) showed broad peaks for the protons close to the Ru(III) center. The broad signal observed at −14.2 ppm is attributed to the *S*-bonded DMSO [40]. ^13^C-NMR spectra of complexes (**1**) and (**2**) are shown in Figures S2f,g, respectively; no peaks were observed in the ^13^C-spectrum of the paramagnetic compound (**2**), due to the effects of paramagnetic broadening. The proposed structures of the complexes are shown in Figure 1.

#### 2.1.4. Electronic Spectra and Magnetic Measurements

The UV-Vis spectrum of Hapbim shows two bands in the 290–341 nm region assigned to π–π* and n–π* transitions, respectively due to the conjugated π system and cyclic azomethine [24,33]. In the complexes, bands in the high-energy region at 302 nm for (**1**) and 289 and 352 nm for (**2**) are assignable to intra-ligand transitions [24]. The diamagnetic complex (**1**) (*d*^6^, *S* = 0) showed two absorption bands at 444 and 612 nm that may arise from Ru(II)-to-ligand (MLCT) and d–d transitions, respectively [41]. The electronic spectra of the paramagnetic complex (**2**) (*d*^5^, *S* = ½, *μ_eff_ =* 1.70 *μ_B_*), exhibits three absorption bands at 419, 498, and 699 nm due to LMCT and d-d transitions, consistent with low-spin octahedral geometry [41,42,43]. Electronic spectra of the ligand and its complexes are shown in Figure S4.

#### 2.1.5. Density Functional Theory Calculations

DFT calculations of compounds (**1**) and (**2**) were performed to confirm their proposed structures (Figure 1, Figure S9 and S10), stability, and electronic properties (Figure S11). Geometry optimizations were performed using the Gaussian 09 program (revision D.01) [44], with the LSDA functional, and the SDD basis set on all atoms as the functional/basis set combination. All calculations employed a polarized continuum model (PCM) for H_2_O. Frequency calculations at the same level of theory confirmed that the optimized structures were located at a minimum on the potential energy surface. For both complexes the calculated structures show modestly distorted octahedral structures, with deviation of dihedral angles around the Ru center less than 10 degrees from the ideal octahedral geometry (Tables S6 and S9). Calculation of the spin density distribution for complex (**2**) (Figure S11, Table S10), demonstrates that the electron spin resides predominantly on the central Ru(III) atom and ligated chlorides. However, significant spin density is also delocalized onto the Hapbim ligands and DMSO, consistent with the paramagnetic shifts and line broadening observed the in the ^1^H-NMR spectrum.

#### 2.1.6. Solution Stability

The solution stability of complexes (**1**) and (**2**) was investigated by UV-Vis spectroscopy in a DMSO-PBS mixture (4% DMSO, 96% PBS) at pH 7.4 over a time period of 24 h (Figure S5a,b). It has been reported that N-heterocyclic ligands can stabilize Ru(II) complexes with respect to aqueous ligand exchange [6,45,46,47,48]. For the Ru(II) complex (**1**) (Figure S5a), it was observed that the bands at 302 and 444 nm remain relatively unchanged over the first two hours, while after 2 and 24 h, the spectrum shows a new broad band at ~715 nm. This process was inhibited when the experiment was repeated with double the concentration of DMSO but was relatively unaffected by the addition of 2 × [Cl^−^], indicating that DMSO exchange predominates (Figure S5b). On the other hand, the Ru(III) complex (**2**) (Figure S5c) showed changes in absorption bands near 288, 326, and 690 nm after 1 h of incubation. Changes in ligand exchange rates were not as prominent in the solution with 2 × [Cl^−^] or 2 × DMSO, but the exchange of chloride ions was slowed somewhat under the former conditions, suggesting chloride hydrolysis (Figure S5d). Similar behavior of the structurally similar complex *mer*-[RuCl_3_(DMSO)(H_2_Biim)] (H_2_Biim = 2,2′-biimidazole) has been reported [49,50].

#### 2.1.7. Cyclic Voltammetry

**Figure 2:**
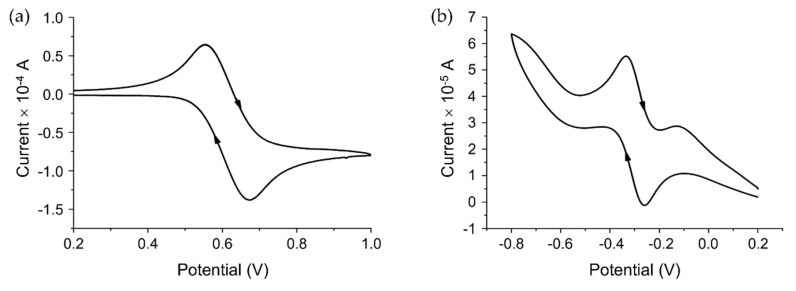
Cyclic voltammograms of (**a**) [Ru(II)Cl_2_(DMSO)_2_(Hapbim)] (**1**) and (**b**) [Ru(III)Cl_3_(DMSO)(Hapbim)] (**2**). Concentration 10^−3^ M, in the CH_2_Cl_2_/DMSO solution with 0.1 M (Bu_4_N)PF_6_, scan rate 50 mVs^−1^.

To study the electrochemical behavior of complexes (**1**) and (**2**), cyclic voltammetry was employed. The cyclic voltammogram of (**1**) (Figure 2a) showed an electrochemically quasi-reversible wave due to the Ru(II)/Ru(III) redox couple [51] (*E*_pa_ = 670 mV, *E*_pc_ = 560 mV and Δ*E* = 110 mV, *E*_1/2_ = 615 mV), and compound (**2**) (Figure 2b) showed an electrochemically reversible redox process [39], (*E*_pa_ = −260 mV, *E*_pc_ = −330 V, Δ*E* = 59 mV and *E*_1/2_ = −295 mV). This indicates that complex (**2**) can be reduced to Ru(II) by biological reducing agents such as ascorbate (*E*° = −50 mV) [52] and glutathione (*E*° = −240 mV) [53]. Although there remains uncertainty regarding the importance of reduction of Ru(III) anticancer complexes to their activity [22], there are a number of observations that suggest such processes may have an impact. For example, KP1019 has an increased affinity for guanine monophosphate (GMP) in the presence of glutathione [54]. Furthermore, a study of Ru(III) indazole complexes showed that cytotoxicity correlated with their reduction potentials [55].

### 2.2. Biological Studies

#### 2.2.1. UV-Vis Studies of DNA Interactions

**Figure 3:**
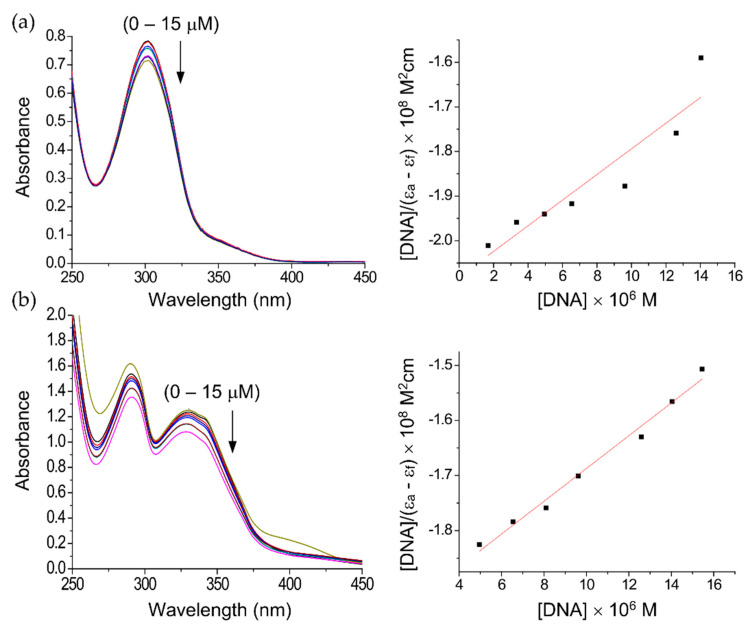
Electronic spectral changes of (**a**) complex (**1**) and (**b**) complex (**2**) in Tris-HCl buffer (pH = 7.4) with increasing concentrations of DNA; [DNA] = 0–15 µM.

The strength and mode of binding of complexes (**1**) and (**2**) to calf thymus (CT) DNA was investigated by UV-Vis spectroscopy. Bands at 302 nm for complex (**1**) and 326 nm for complex (**2**) were monitored to report changes in absorbance with increasing concentrations of DNA. It was observed that for both complexes these bands exhibit concentration-dependent hypochromism without any shift of their maximum absorbance (Figure 3a,b).

This is consistent with the complexes binding to DNA via intercalation [56], which has been observed previously for Ru(II)-DMSO complexes [48,57]. The intrinsic DNA binding constants (K_b_) determined from these data were K_b_ = 1.38 ± 0.05 × 10^4^ M^−1^ for complex (**1**) and K_b_ = 1.68 ± 0.02 × 10^4^ M^−1^ for complex (**2**), which are higher than the free ligand (1.26 ± 0.03 × 10^4^ M^−1^). These values are smaller than the structurally related Ru(II)-DMSO complexes (2.21–2.49 × 10^5^ M^−1^) [48,57], but significantly larger than reported for a 2-(2-naphthoimidazole)-1,10-phenanthroline ruthenium(II) complex (1.6 × 10^3^ M^−1^) [58]. Based on these comparisons, the values for (**1**) and (**2**) may indicate binding to DNA by partial intercalation.

#### 2.2.2. Fluorescence studies of DNA interactions

**Figure 4:**
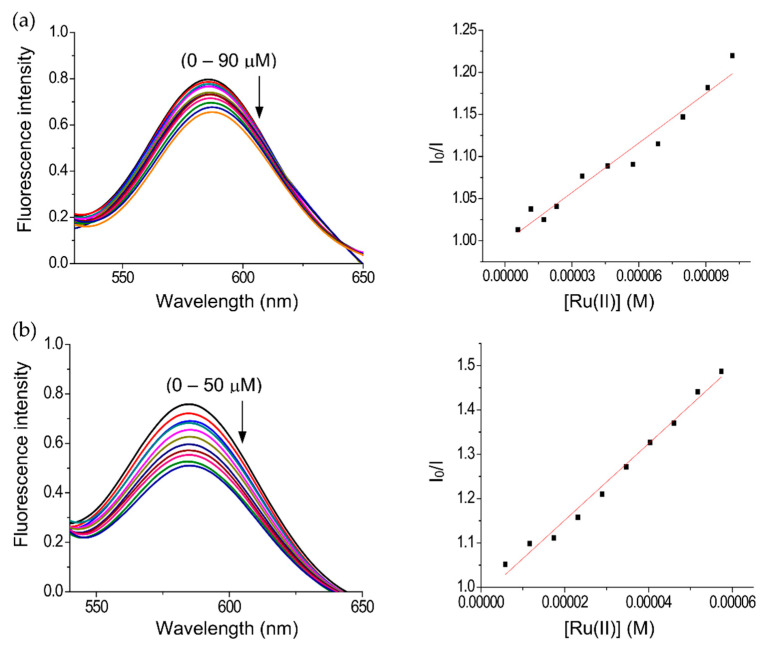
Fluorescence quenching spectra of ethidium bromide-DNA (EB-DNA), (**a**) complex (**1**), and (**b**) complex (**2**), [DNA] = 5 μM, [EB] = 5 μM and compounds (5–90 μM) for complex (**1**) and (5–50 μM) for complex (**2**).

The binding affinity of the complexes towards CT DNA was also studied using an ethidium bromide (EB-DNA) assay (Figure 4a,b). Ethidium bromide is weakly fluorescent in buffer [59,60,61], but shows a strong fluorescent emission upon intercalative DNA binding [48,62]. Thus, the addition of competitive DNA binding molecules attenuates the EB emission by competition for binding sites [63,64]. This behavior was observed for (**1**) and (**2**), with incremental addition of the complexes to the EB-DNA adduct, resulting in a concomitant reduction in fluorescence intensity. These observations indicate displacement of EB by intercalation or partial intercalation [57,64]. Complex (**2**) showed a better affinity towards DNA than complex (**1**), consistent with the data obtained from the UV-vis titrations (see above). The quenching constant (K_q_) and the apparent binding constant (K_app_) values (Table 1) are higher than other related ruthenium complexes [48,65], suggesting that these interactions are relevant to the cytotoxic activity of the compounds.

### 2.3. Anticancer Activity

#### 2.3.1. In Vitro Assays

##### Effect of Hapbim Ligand and Its Complexes (**1**) and (**2**) on MCF7, Caco2, and THLE-Cell Viability

MTT assays were performed to evaluate the effect of both the Hapbim ligand and complexes (**1**) and (**2**) on the proliferation of human MCF7 and Caco2 cancer cells and THLE-2 normal human liver cells (Table 2, Figure S6). Cisplatin was used as a positive control. Cells were treated with varying concentrations of each compound, and all tested compounds, as well as cisplatin, exhibited significant dose-dependent anti-proliferative activity. Complex (**2**) showed higher activity against the MCF7 and Caco2 cell lines (IC_50_ = 230 ± 10 and 250 ± 10 µM, respectively) than both (**1**) (IC_50_ = 320 ± 20 and 290 ± 20 µM), and the Hapbim ligand (IC_50_ = 290 ± 20 and 380 ± 20 µM), but the IC_50_ values of the complexes indicate a lower in vitro cytotoxic effect than cisplatin [66]. The enhanced cytotoxicity of (**2**) may be related to activation by reduction of the Ru(III) center in the comparatively reducing environment of the cancer cells [13,67]. Significantly, all three compounds (ligand, (**1**) and (**2**)) exhibited minimal cytotoxic effects on normal THLE-2 cells (Table 2). The ratio of the cytotoxicity against THLE-2 cells to the MCF7 and Caco2 cell lines for Hapbim, the complexes (**1**), (**2**), and cisplatin were used to calculate the therapeutic index (TI). As shown in Table 3, the TI of compound (**2**) was approximately two-fold higher than the TI of (**1**), further suggesting that (**2**) has the greatest potential as a cancer-targeting treatment. However, the TI of cisplatin was found to be higher than either (**1**) or (**2**), despite the platinum drug having significant known side effects in vivo [68,69].

At first glance, the comparison of the in vitro cytotoxic properties of the complexes with cisplatin might initially suggest that they are not particularly favorable chemotherapeutic candidates. However, it has been shown that in vitro studies can be poor predictors of the in vivo activity of Ru anticancer complexes [70]. Furthermore, the in vitro TI serves only as a crude indicator of compound toxicity, with TIs determined against several types of normal cells originating from different organs required to give a better picture of toxicity [71]. As we show in this report, in vivo studies (see below) can be critical in assessing the true chemotherapeutic potential of Ru complexes and their mechanism of action, and for evaluating off-target toxicity towards specific organs [72]. Due to its superior activity, complex (**2**) was selected for further DNA fragmentation analysis, in vitro cell-cycle experiments, and in vivo studies.

##### Compound (**2**) Inhibits Proliferation of MCF7 and Caco2 Cells via Induction of Apoptosis

**Figure 5:**
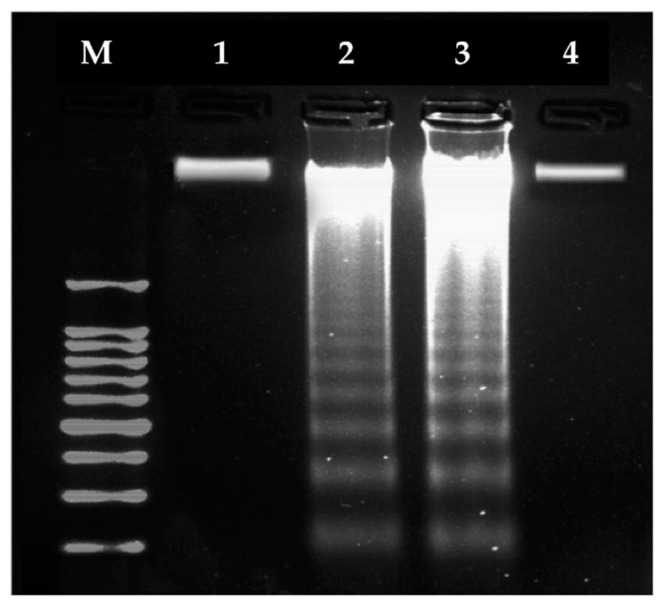
Effects of compound (**2**) treatment on DNA fragmentation in MCF7 and Caco2 cells as compared to untreated (control) cells. **Lane 1**: Untreated MCF7; **Lane 2**: Complex (**2**)-treated MCF7; **Lane 3**: Complex (**2**)-treated Caco2; **Lane 4**: Untreated Caco2 cells; **M**: 100 bp marker.

Inhibition of cancer cell proliferation via induction of apoptosis (programmed cell death) is a desirable mechanism for anticancer drugs [73]. This can be analyzed by DNA fragmentation, which is an indicator of apoptosis [74], and was studied for compound (**2**) with MCF7 and Caco2 cells. Treatment with compound (**2**) resulted in distinct DNA laddering in both cancerous cell lines, whereas untreated control cells showed normal, intact DNA (Figure 5). This induction of DNA fragmentation by (**2**) suggests that the complex may trigger cancer cell death via, at least in part, induction of apoptosis. In addition, since DNA laddering was observed in two divergent (unrelated) cancer cell lines, the anticancer effect of (**2**) is likely not specific to specific cancer cell types.

##### Effect of Compound (**2**) on Cell-Cycle Arrest in MCF7 and Caco2 Cells

**Figure 6:**
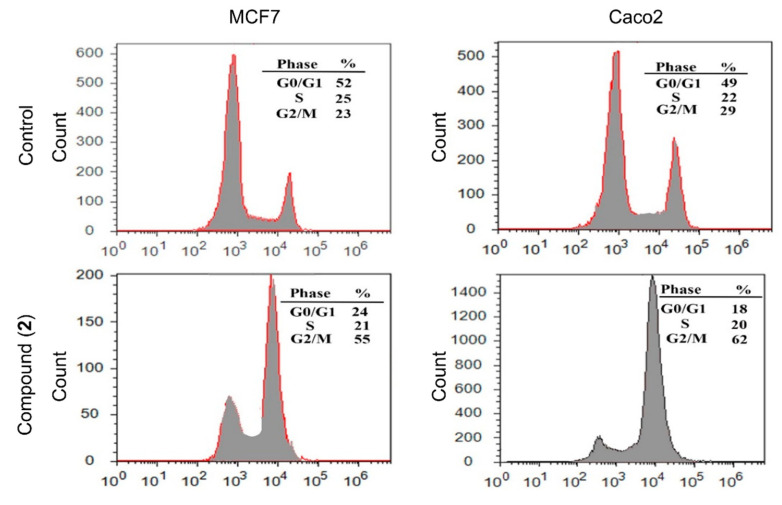
Effect of compound (**2**) on % of cells in the three cell-cycle phases of MCF7 and Caco2 cells as measured by flow cytometry. The *x*-axis represents the propidium iodide (PI) fluorescence based on the DNA content and the *y*-axis represents the number of cells in each phase.

When cells are exposed to stressors, check-point signals are activated in the G1/S or G2/M phase, resulting in cell-cycle arrest and apoptosis [75]. The effect of complex (**2**) on MCF7 and Caco2 cell cycles was determined by flow cytometry using the PI stain to evaluate DNA content. Treatment with (**2**) led to a significant elevation in the number of cells in the G2/M phase as compared to untreated cells: MCF7, control = 23%, treated = 55%; Caco2, control = 29%, treated = 62% (Figure 6). This treatment also resulted in a reduction in the number of MCF7 and Caco2 cells in the G0/G1 phase: MCF7, control = 52%, treated = 24%; Caco2, control = 49%, treated = 18%. No significant change in the cell numbers in the S phase was observed. Increased numbers of MCF7 and Caco2 cells in the G2/M phase indicates that compound (**2**) may induce apoptosis in these two cell lines by triggering cell-cycle arrest in this phase. This observation combined with the DNA fragmentation studies suggests that complex (**2**) may target the checkpoint between cell-cycle progression and apoptosis.

##### Effect of Compound (**2**) on Ehrlich Carcinoma Cell Viability

Based on the IC_50_ values of the complexes (**1**) and (**2**) against the MCF7 and Caco2 cell lines, as well as the in vitro DNA binding assay, compound (**2**) was selected for testing against Ehrlich ascites carcinoma (EAC) cells for additional in vitro and in vivo experiments. The in vitro studies used a viability exclusion test with trypan blue dye. As expected, increasing the concentration of (**2**) led to increased Ehrlich cell death in a dose-dependent manner (Figure S7). The IC_50_ of (**2**) against EAC cells was determined to be 70 ± 1 μg/mL, which compares well, for example, with the anticancer drug methotrexate (IC_50_ = 98 ± 2 μg/mL) [76].

#### 2.3.2. In Vivo Experiments

Examination of the effect of different concentrations of compound (**2**) on several mice groups determined that the LD_50_ value was 60 mg/kg. To determine the maximum tolerated dose by mice; four healthy groups (five mice in each) were intraperitoneally injected with 6 (1/10 LD_50_), 12, 18, 30, and 40 mg/kg for 12 days. These mice were examined daily for any sign of toxicity and weighed every two days. The 30 and 40 mg/kg doses were toxic after 3-5 days and all the animals were dead on the fifth day. No weight loss or signs of toxicity were noticed in the other groups until the 12th day. Therefore, doses of 6, 12, and 18 mg/kg were used in subsequent experiments.

To evaluate the in vivo activity of compound (**2**), four groups of mice were studied: (i) Control group, healthy normal mice were intraperitoneally injected with vehicle; (ii) I, II, and III control groups, healthy normal mice were intraperitoneally injected with 6, 12, and 18 mg/kg of the complex; (iii) EAC group, mice were intraperitoneally injected with a single dose of EAC cells, (iv) I, II, and III treated groups, mice were intraperitoneally injected with a single dose of EAC cells, and intraperitoneally injected with 6, 12, and 18 mg/kg Ru(III) complex, respectively. Injections of vehicle and/or compound (**2**) were carried out for 12 days.

**Figure 7:**
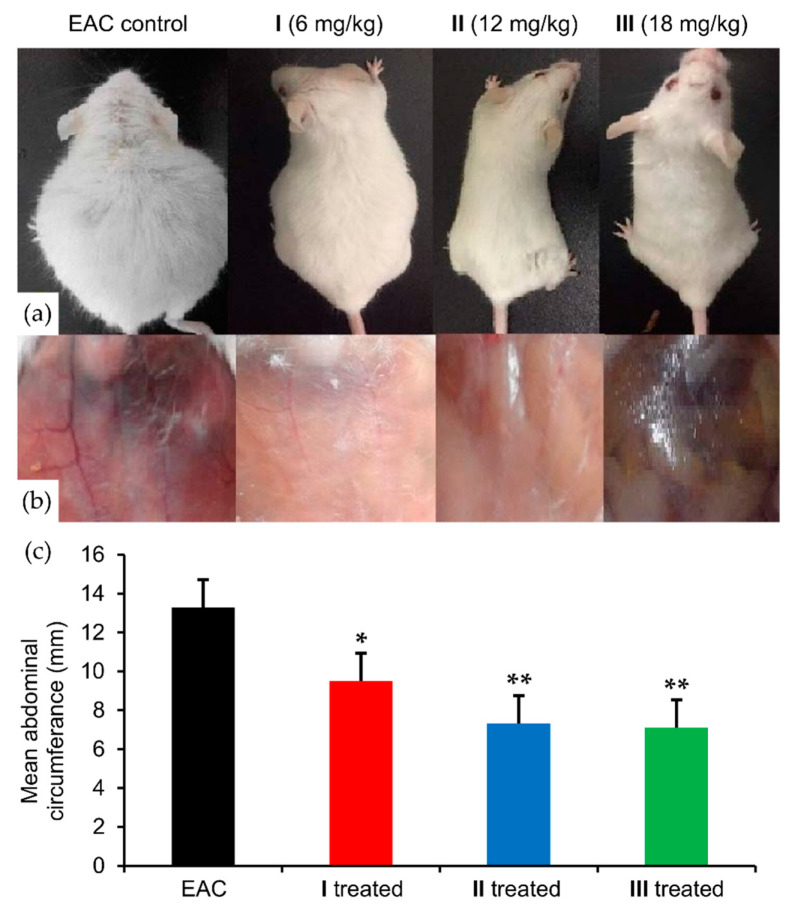
Effect of compound (**2**) on abdominal size and peritoneal angiogenesis. (**a**) From left to right Ehrlich Ascites Carcinoma (EAC), **I** (6 mg/kg), **II** (12 mg/kg), and **III** (18 mg/kg) treated mice; (**b**) from left to right, the peritoneal cavity of EAC, **I**, **II**, and **III** treated groups; (**c**) mean abdominal circumference of EAC, **I**, **II**, and **III** treated groups. Values (columns and error bars) are expressed as mean ± SEM. * and ** mean significant difference at *p* < 0.05 and *p* < 0.001 compared to the EAC group.

EAC is a liquid tumor that spreads and proliferates in the abdominal region of the inoculated mice. Consequently, the untreated EAC group of mice had a larger mean abdominal circumference (13.20 ± 0.03 cm) than the control group (7.06 ± 0.18 cm). Treatment with (**2**) led to a dose-dependent reduction in the mean abdominal circumference of the EAC treated groups, **I** (6 mg/kg), **II** (12 mg/kg), **III** (18 mg/kg) with values of 9.6 ± 0.2, 7.30 ± 0.03, and 7.10 ± 0.03 cm, respectively. These results demonstrate that all three dose regimens of (**2**) were sufficient to stop EAC proliferation (Figure 7a), with the highest dose reducing the mean abdominal size to the control value.

Angiogenesis is a process by which cancer cells can maintain their rapid proliferation [77] through the construction of a new vascular network to increase the flow of oxygen and nutrients to the cancer site and remove waste products [78]. The new blood vessels also can allow the primary cancer to leak from its site into the blood stream and invade other organs leading to metastasis [79]. An indication of angiogenesis in the inner peritoneal lining of EAC mice has been approved as an in vitro angiogenesis assay [80]. Using this approach after sacrificing, as described by Saraswati et al. [81], we found increased angiogenesis in the EAC bearing mice. Significantly, in the treated groups angiogenesis was decreased in a dose-dependent manner, with the lowest effect for the smaller dose (Figure 7b). These findings suggest that (**2**) not only kills cancer cells but also may prevent metastasis through inhibition of angiogenesis.

To study the intrinsic toxicity of compound (**2**), tests were performed of liver and kidney function by evaluating serum levels of biochemical markers (Table 4) following the treatment of normal healthy mice with the complex. Hepatocytes produce liver enzymes such as glutamate-pyruvate transaminase (GPT) and glutamate-oxaloacetate transaminase (GOT), in minute quantities to begin the transamination reactions in amino-acid metabolism. When liver inflammation occurs because of hepatocyte damage, injury, or cancer, these enzymes are released in large quantities into the blood stream leading to increased levels in the plasma. Elevation of GPT and GOT levels correlates with the severity of liver damage [82], and following healing or repair of hepatocytes, these enzyme levels typically decrease again.

The toxicity of compound (**2**) was initially evaluated in healthy mice by analyzing levels of several biological markers at **I** (6 mg/kg), **II** (12 mg/kg), and **III** (18 mg/kg) doses of the compound (Table 4). Treatment with (**2**) caused some elevation of GPT at the higher concentrations, consistent with minor toxicity towards hepatocytes. However, this toxicity is not expected to cause severe liver damage since GPT serum levels were similar to the healthy control group. The highest concentration of (**2**) also resulted in moderately elevated bilirubin and creatinine levels. Overall, these results demonstrate that the compound exhibits relatively minor hepatotoxicity and nephrotoxicity at the doses tested.

A single dose of cisplatin can cause nephrotoxicity in experimental animals [68]. By contrast, compound (**2**) was injected for 12 days with a little sign of kidney toxicity (the creatinine level was only increased at high dose) and normal levels of urea and uric acid. These data are similar to that reported for [RuCl(AMBI)(H_2_O)_3_]Cl_2_ (HAMBI = 2-aminomethyl benzimidazole), demonstrating the general potential for ruthenium anticancer complexes to be safe towards the renal system [29,83].

Compared to the normal healthy mice, the EAC mice exhibited highly elevated GPT and GOT. Treatment with compound (**2**) decreased the levels of both of these markers significantly in a dose-dependent fashion. However, even at the highest dose (18 mg/kg), GOT levels were still greater than in the healthy mice (Table 4). GPT levels returned to control levels at the lower concentrations (6, 12 mg/kg), but were elevated somewhat at the highest dose, suggesting the effect of intrinsic toxicity of the compound. In untreated EAC mice, albumin, bilirubin, creatinine, urea, and uric acid were all elevated. With the exception of creatinine, which was not strongly affected, levels of all these species were essentially returned to those found in the normal healthy mice following treatment with (**2**). In the case of bilirubin, urea, and uric acid this was observed even at the lowest treatment level (6 mg/kg). Taken together, these observations are very encouraging in the context of chemotherapeutic potential of compound (**2**) since they demonstrate removal of the cancer burden with minimal off-target toxicity.

##### Antioxidant Activity Assay

Cancer cells frequently have elevated metabolic activity due to high proliferation rates. This leads to increased waste products and raises levels of reactive oxygen species (ROS) [84], resulting in damage to tissues, organs, and DNA [85]. To compensate for the elevated ROS, cells release antioxidant enzymes [86]. Important scavengers of ROS include superoxide dismutase (SOD), which protects the cell from superoxide anion (O_2_·^-^) damage, generating hydrogen peroxide [87], and catalase (CAT), which scavenges intracellular hydrogen peroxide (H_2_O_2_) to further reduce oxidative cell damage [88]. Additional antioxidant activity is provided by reduced glutathione (GSH), which deactivates ROS and radical species [89]. The total antioxidant capacity (TAC) measures the overall amount of free radicals scavenged in the biological system [90].

**Figure 8:**
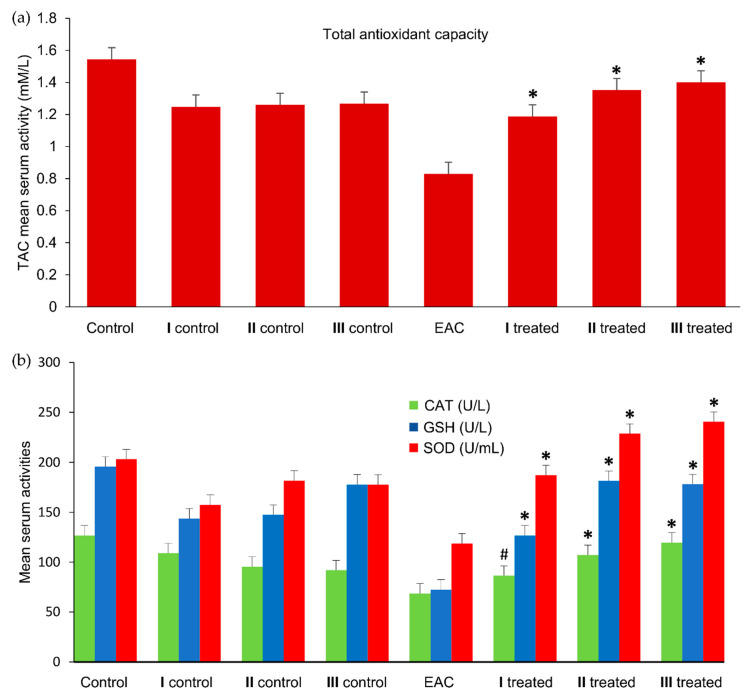
(**a**) Total antioxidant activity (TAC) and (**b**) catalase (CAT), superoxide dismutase (SOD), glutathione (GSH) activity in sera of healthy control, EAC, and treated groups. Values (columns and error bars) are expressed as mean ± SEM. # Indicates significant difference at *p* > 0.05, * indicates significant difference at *p* > 0.0001 as compared to the untreated EAC group.

The higher level of ROS in cancer cells is correlated with increased activity of oxidases, cyclooxygenases, and lipoxygenases [91]. To protect cell mitochondria from ROS, excess SOD is released [92]. Since SOD has a role in preventing mitochondrial DNA damage this can enable normal cells to recover from the effects of ROS [92]. As shown in Figure 8b, the SOD levels in the untreated EAC group were reduced compared to the healthy control group (*p* < 0.0001), but were elevated in **I** (6 mg/kg), **II** (12 mg/kg), and **III** (18 mg/kg) treated groups as compared to the untreated EAC group (*p* < 0.02 and *p* < 0.0001 and *p* < 0.0001, respectively). This increase may be due to the inhibition of EAC cell proliferation by (**2**) and excessive SOD release to scavenge the superoxide anion produced by damaged EAC cells. Our hypothesis is that the Ru(III) complex (**2**) not only stops the growth of the inoculated EAC cells but also causes cell death. These dead cancer cells may then release their intracellular ROS.

The levels of GSH and CAT also were significantly reduced in the untreated EAC group when compared with their levels in the healthy control group, but were increased in the three treated groups as compared to the untreated EAC group values (Figure 8b). These results confirm the reduction of cancer burden by (**2**) and the repairing of the cells by their antioxidant system. Cancer cells can invade organs and can cause inflammation of normal cells [93]. Following the death of cancer cells, such as from the action of an anticancer drug, antioxidant enzymes can repair inflamed cells enabling them to normally proliferate [94]. Correspondingly, the total antioxidant capacity (TAC) (Figure 8a) was decreased in the untreated EAC group as compared to the normal control group (*p* < 0.0001), but significantly increased in the three treated groups (*p* < 0.0001). In general, the in vivo studies demonstrate that compound (**2**) can promote the antioxidant activity by increasing levels of SOD, GSH, and CAT. This leads to an overall increase in antioxidant capacity, reducing cell concentrations of ROS.

##### Effect of Complex (**2**) on the Expression of Apoptosis-Related Genes in the Liver of Ehrlich Mice

**Figure 9:**
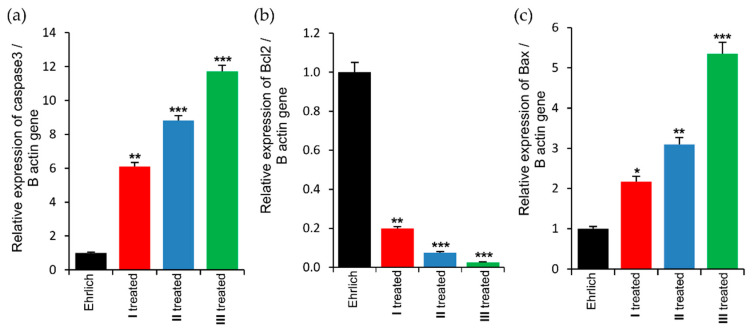
Real-time quantitative PCR analysis of the (**a**) caspase 3, (**b**) *Bcl2*, and (**c**) *Bax* gene expression in liver tissue of Ehrlich mice. Analyses were performed in triplicate. Values (columns and error bars) are expressed as mean ± SEM. *, **, and *** indicated significant difference at *p* < 0.05, *p* < 0.001, and *p* < 0.0001, respectively.

Apoptosis normally occurs in a sequence of steps to stimulate and/or suppress certain genes and proteins, and ends with protease activation, DNA fragmentation, and cell death [95]. Cancer cells can inhibit apoptosis and tolerate apoptotic signals, and the majority of chemotherapeutic agents act by enhancing apoptotic pathways in cancer cells. Genes such as the *p53* and *Bcl2* families play a dual role in cell division as well as in apoptosis, and overexpression of these genes may encourage cell proliferation, cell-cycle arrest, or cell death. The *Bcl2* gene family products have two main roles; antiapoptotic (inhibit cell death) via the *Bcl2* protein and proapoptotic (support cell death) via *Bax* [96]. *Bcl2* controls apoptosis and enhances cancer-cell survival and thus promotes resistance to chemotherapeutic agents [97]. By contrast, *Bax* is a proapoptotic gene that supports cell death by increasing mitochondrial membrane permeability and inhibiting cell proliferation [98]. The last signaling enzyme in these apoptotic pathways is caspase 3, which triggers DNA degradation leading to cell death. In normal cells, apoptotic pathways involve *Bcl2* inhibition and *Bax* activation, and terminate with proteolytic enzyme (caspase 3) regulation, which leads to cell lysis and death [98,99]. EAC cells spread and proliferate in hepatocytes causing hepatocellular dysfunction [100], which leads to an increase in the antiapoptotic factors *Bcl2* and a decrease in proapoptotic *Bax* gene expression [101]. As shown in Figure 9, real-time quantitative PCR (qPCR) of liver tissue from EAC-bearing mice demonstrates that treatment with compound (**2**) reverses these effects, with a significant dose-dependent reduction of *Bcl2* and recovery of *Bax* gene expression [102]. The qPCR studies revealed significant increases in the mRNA levels of both apoptotic markers, *Bax* and caspase 3, following treatment, as compared to untreated Ehrlich mice, with the strongest effect at higher dose (Figure 9). By increasing the expression of caspase 3, DNA damage followed by lysis of the EAC cells could be initiated, leading to apoptotic cell death following treatment with (**2**) [103]. This is consistent with the study of Bahar et al., which demonstrated that caspase 3 signals were increased in the liver of EAC groups treated with the anticancer candidate silibinin, as compared to an untreated EAC group [101]. Thus, complex (**2**) likely suppresses cancer-cell proliferation in the liver by induction of apoptosis, as revealed by the enhancement of *Bax* and caspase 3 levels and reduction of *Bcl2*.

## 3. Materials and Methods

### 3.1. Materials

The starting materials, RuCl_3_ × H_2_O (Pressure Chemical, PA, USA), 2-aminophenyl benzimidazole (Alfa Aesar, Haverhill, MA, USA), calf thymus DNA (Type XV, activated lyophilized powder and ethidium bromide (Sigma-Aldrich)) and HPLC grade solvents (Alfa Aesar) were used as received. *Cis*-[RuCl_2_(DMSO)_4_] [34] and [DMSO)_2_H][*trans*-RuCl_4_(DMSO)_2_] [104] were prepared as reported in the literature. The Ehrlich ascites carcinoma (EAC) cell line, as a model of mammary gland carcinoma, was obtained from the National Research Center (NRC), Dokki, Egypt. Human breast cancer (MCF7), human colorectal cancer (Caco2), and human normal liver (THLE-2) cell lines were obtained from American Type Culture Collection (ATCC, Manassas, VA, USA). Serum glutamate-pyruvate transaminase (GPT), serum glutamate-oxaloacetate transaminase (GOT), albumin, total bilirubin, creatinine, urea, and uric acid kits were obtained from Spinreact, Barcelona, Spain. Superoxide dismutase (SOD), reduced glutathione (GSH), catalase (CAT), and total antioxidant capacıty (TAC) kits were purchased from Biodiagnostic, Dokki, Egypt. Fetal bovine serum, penicillin/streptomycin, and Dulbecco’s modified Eagle’s medium (DMEM) were obtained from GIBCO, MD, USA. MTT (3-(4,5-dimethylthiazol-2-yl)-2,5-diphenyltetrazolium bromide) was purchased from Sigma. RNeasy Mini kit (QIAGEN, Germantown, MD, USA), Quantiscript reverse transcriptase, and QuantiTect SYBR Green qPCR Master Mix were obtained from QIAGEN, Germantown, MD, USA.

### 3.2. Measurements

Elemental analyses (C, H, N) were performed by the Microanalysis unit in the Department of Chemistry at Simon Fraser University. Infrared spectra (FTIR) were performed on a JASCO 4100 FTIR spectrophotometer in the 4000–400 cm^−1^ range using a KBr disc. NMR (^1^H and ^13^C) spectra were recorded in DMSO-d_6_ solvent using a Bruker 400 MHz instrument. The 2D NMR analysis (^1^H–^1^H COSY) was performed on a Jeol 500 MHz NMR, at Mansoura University. ESI/APCI HRMS data were acquired on a Thermo Scientific Exactive Plus Orbitrap mass spectrometer. Electronic spectra (in DMSO, 200–900 nm) were measured on a JASCO 630 V spectrophotometer. Emission spectra of the complexes were measured on a Jenway model 6285 spectrofluorimeter for excitation and emission spectra, 200–700 nm. The magnetic susceptibility of the complexes was measured on a Johnson Matthey magnetic susceptibility balance. Cyclic voltammetry was studied in a DMSO/CH_2_Cl_2_ solution containing a supporting electrolyte, (Bu_4_N)PF_6_, using a CH Instruments 610D electrochemical analyzer equipped with a three-electrode cell composed of silver and platinum wires as reference and working electrodes, respectively. A microplate reader (StatFax-2100, Awareness Technology, Inc., Palm City, FA, USA), Attune flow cytometer (Applied Biosystems, Foster City, CA, USA), Quawell nanodrop Q5000 (San Jose, CA, USA), StepOnePlus real time PCR system (Applied Biosystems, Foster City, CA, USA), and CO_2_ incubator (NuAire In-Vitro Cell, Plymouth, MN, USA) were used in biological studies.

### 3.3. Synthesis

#### 3.3.1. [Ru(II)Cl_2_(DMSO)_2_(Hapbim)] (**1**)

2-aminophenylbenzimidazole (Hapbim), (0.25 mmol, 0.052 g) was dissolved in 10 mL of ethanol and *cis*-[RuCl_2_(DMSO)_4_] (0.25 mmol, 0.121 g) was added. The reaction mixture was stirred under reflux for 2 h. Initially, the solution turned green, followed by production of a yellow precipitate. The resulting product was filtered off, washed with acetone and ether, then dried in vacuo. Yield, 66%; mp > 200 °C; Anal. Calc. for C_17_H_23_C_l2_N_3_O_2_RuS_2_ is calculated: C, 37.99; H, 4.31; N, 7.82%. Found: C, 38.09; H, 4.54; N, 7.91%. FTIR (KBr, cm^−1^): ν(NH_2_) 3251 (s), ν(NH) 3113 (s), ν(C=N) 1626 (m), ν(SO) 1051 (s), ν(Ru-N) 441 (s). ^1^H-NMR (DMSO-d_6_, δ ppm): 13.53 (s, N*H*, 1H), 7.89 (d, ^3^*J* = 6.9 Hz, **4**, 1H), 7.76 (d, ^3^*J* = 8.9 Hz, **3′**, 1H), 7.61 (d, **7**, ^3^*J* = 6.7 Hz, 1H), 7.38 (t, ^3^*J* = 7.6 Hz, **6′**, 1H), 7.37–7.29 (m, **5**, **6**, 2H), 7.27 (t, ^3^*J* = 7.5 Hz, **5′** 1H), 7.16 (m, **4′**,2H), 6.18 (d, ^2^*J* = 8.7 Hz, N*H*, 1H), 3.02, 3.23, 3.34 (coincident with H_2_O signal), 3.48 (s, C*H_3_*-DMSO). ^13^C NMR (101 MHz, DMSO) δ 151.7, 143.5, 142.7, 134.2, 130.4, 127.6, 124.5, 124.4, 123.3, 122.2, 121.8, 120.5, 112.0, 45.9, 45.8, 43.0, 42.9. HRES-MS (Calcd, Found. *m/z*): 535.97 (535.96, [RuCl_2_(DMSO)_2_(Hapbim)]-H]^−^) (Figure S3a). UV-Vis (4 × 10^−5^ M, DMSO): λ_max_ (nm), (ε, L. mol^−1^, cm^−1^): 302 (ε = 19250) and 444 (997.5), 612 (575).

#### 3.3.2. [Ru(III)Cl_3_(DMSO)(Hapbim)]·H_2_O (**2**)

[DMSO)_2_H][*trans*-RuCl_4_(DMSO)_2_] **(1)** (0.25 mmol, 0.139 g) was dissolved in anhydrous ethanol (4 mL) then warmed up to fully solubilize the compound. Then, this was added to a 4 mL anhydrous ethanolic solution of 2-aminophenylbenzimidazole (0.25 mmol, 0.052 g), which was slightly warmed to improve solubility. The reaction contents were then stirred at approximately 40 °C for 2 h. This solution was then concentrated in vacuo to an approximate volume of 1 mL, to which 15 mL of diethyl ether was added, resulting in a dark green precipitate. This precipitate was then filtered and washed with cold diethyl ether and cold ethanol, then subsequently dried to yield **2**. Yield: 62; mp > 200 °C. Anal. Calc. for C_15_H_19_Cl_3_N_3_O_2_RuS is calculated: C, 35.13; H, 3.73; N, 8.19%. Found: C, 35.36; H, 3.95; N, 8.24%. FTIR (KBr, cm^−1^): ν(NH_2_) 3239 (s), ν(NH) 3101 (s), ν(C=N) 1620 (m), ν(SO) 1091 (s), ν(Ru–N) 445 (m). ^1^H-NMR (DMSO-d_6_, δ ppm): 6.7 (bs), 10.9 (bs), 9.7 (bs), −14.2 (bs, CH_3_-DMSO, 6H). HRES-MS (Calcd, Found. *m/z*): 494.91 (494.92, ([RuCl_3_(DMSO)(Hapbim)]-H]^−^)) (Figure S3b). UV-Vis (4 × 10^−5^ M, DMSO): λ_max_ (nm), (ε, L. mol^−1^, cm^−1^): 289 (23375), 353 (18775), 419 (6475), 498 (1250), 669 (875).

### 3.4. Solution Stability

Stability studies of complexes (**1**) and (**2**) were carried out by UV-Vis spectroscopy in the 200–900 nm range. A 1 × 10^−4^ M solution of each complex was prepared by dissolving the complexes first in DMSO (to improve solubility), and was then completed to the required volume with PBS (phosphate buffered saline, PBS, pH 7.4 containing 137 mM NaCl, 2.7 mM KCl, 10 mM Na_2_HPO_4_, and 1.8 mM K_2_HPO_4_) to give (4% DMSO/PBS) solution. The electronic spectra for the solutions were measured over 24 h.

### 3.5. Biological Studies

#### 3.5.1. DNA Interactions

##### UV-Visible Spectra

To determine the DNA binding mode and binding constant of the metal complexes, CT DNA interactions were studied by UV-Vis titration. In these experiments, the electronic spectra of the complexes were measured in Tris-HCl buffer (5 mM Tris-HCl and 50 mM NaCl, pH 7.4) in the presence of different concentrations of CT DNA. The CT DNA concentration was determined spectrophotometrically at 260 nm (ε = 6600 M^−1^cm^−1^). To prepare the stock solution of each complex (12.5 µM) they were first dissolved in a minimum amount of DMSO, then the required concentration was obtained by dilution with tris-HCl buffer. Different concentrations of CT DNA (0–15 µM) were added (an equivalent amount of DNA was added to the reference cell to eliminate the absorbance of DNA itself). After a 5 min incubation period, the absorption spectra were recorded in the range 200–600 nm. The binding constants (*K_b_*) of the complexes were calculated using Equation (1).

(1) $$\frac{\left[\mathrm{DNA}\right]}{\left(\varepsilon_{a}-\varepsilon_{f}\right)}=\frac{\left[\mathrm{DNA}\right]}{\left(\varepsilon_{b}-\varepsilon_{f}\right)}+\frac{1}{K_{b}(\varepsilon_{b}-\varepsilon_{f})}$$

where [DNA] is the molar concentration of base pairs, and *ε_a_*, *ε_f_*, *ε_b_* correspond to the apparent extinction coefficient of the bound complex (A_obs_/[compound]), the extinction coefficients of the free complex, and the extinction coefficient of the fully bound complex or ligand in the solution, respectively [105]. Plotting [DNA]/(*ε_a_* − *ε_f_*) vs. [DNA] gives the value of *K_b_* (the ratio of slope to intercept).

##### Fluorescence Spectra

The binding affinity of the metal complexes towards CT DNA was evaluated using competitive fluorescence studies. CT DNA pretreated with ethidium bromide (EB) ([DNA] = [EB] = 5 μM) in Tris-HCl/NaCl buffer (pH = 7.2) was incubated with the complexes in concentrations of 0–90 μM. After a 3-min incubation period, the reduction of emission intensity was measured; the complexes do not exhibit fluorescence emission. The quenching of EB-DNA adduct fluorescence was followed by monitoring the emission spectra in the wavelength range of 530–650 nm. Upon addition of the metal complexes to the EB-DNA adduct, the quenching binding constant (K_q_) at λ = 585 nm is determined from the Stern-Volmer Equation (2) [106]:

I_0_/I = 1 + K_q_[Q] (2)

where I_0_ and I are the fluorescence intensities of EB-DNA in the presence and absence of the metal complexes, respectively, [Q] is the concentration of the tested compounds, and K_q_ is the quenching constant calculated from the slope of the straight line obtained from plotting I_o_/I vs. [Q]. In addition, the apparent binding constant can be calculated from Equation (3):

K_EB_[EB] = K_app_[Q] (3)

where K_EB_ = 1 × 10^7^ M^−1^ [EB] is the ethidium bromide concentration (5 μM) and [Q] is the concentration of the quencher that provides a 50% intensity reduction of the initial EB-DNA adduct.

### 3.6. In Vitro Assays

#### 3.6.1. Cell Viability by MTT Assay

The cytotoxic effect of the Hapbim ligand and its complexes (**1**) and (**2**) on human breast cancer (MCF7), human colorectal cancer (Caco2), and human normal liver (THLE-2) cell lines was evaluated by the MTT assay. The cells were cultured in a 96-well plate (1 × 10^4^ cells/well, 100 μL/well) containing DMEM medium provided with 10% fetal bovine serum, and 1% penicillin/streptomycin. Then, the cells were incubated at 37 °C for 24 h in a CO_2_ incubator under 5% CO_2_, 95% air until reaching a confluence of 70–80%. A 20 μL volume of each compound at different concentrations (from 5 to 400 μM for the cancer cell and from 600 to 1000 μM for the normal cells) was added and the cells were re-incubated for a further 24 h. After the incubation period, the cells were incubated again with 5 mg/mL of MTT for 4 h and then the medium was replaced with 100 μL DMSO and vortexed for 20 min. Finally, the absorbance was recorded at 570 nm using the microplate reader. The concentration of the compounds inhibiting 50% of cells (IC_50_) was calculated by fitting the sigmoidal dose-response curves to a four-parameter logistic model using the GraphPad (Prism 7) statistical software (Inc., La Jolla, CA, USA). To calculate the cell inhibition percentage, the following equation was used:

% Cell inhibition = 100 − % cell viability ((A_t_ − A_b_)/(A_c_ − A_b_)) × 100%) (4)

where A_t_ is the absorbance value of tested compound; A_b_ is the absorbance value of blank; A_c_ is the absorbance value of the control.

#### 3.6.2. EAC Cytotoxicity Assay (In Vitro)

The cytotoxicity determination of compound (**2**) against EAC cells was calculated by the trypan blue exclusion method [107]. The EAC cells were aspirated from the mouse’s peritoneal cavity by a sterile syringe and immediately transferred into a sterile falcon tube. Then, the cells were washed with sterile PBS to remove any serum or blood traces and centrifuged for 3 min, after which the supernatant was removed. After washing 2–3 times with PBS, the cell pellet was aspirated into a sterile falcon tube and every 1 mL of cell pellet was resuspended in 9 mL of sterile PBS until a concentration of 10^10^ cells in 0.1 mL was achieved, as determined with a hemocytometer; where 0.1 mL of cell suspension was added to 0.880 mL PBS then 20 μL of trypan blue was added. The cells’ viability was checked using a hemocytometer and found to be greater than 98%. A volume of 10 μL of different concentrations of compound (**2**) (5 to 400 µM) was added to 0.1 mL of cell suspension and the tested volumes were adjusted to 980 μL with PBS, then incubated for 2 h at 37 °C. In a control test tube, 10 μL of solvent (2% DMSO) was added to a 0.1 mL cell suspension and 870 μL of PBS. After incubation, 20 μL of trypan blue was added to each tube, mixed for 30 s, then 10 μL of solution from each tube was injected under the hemocytometer coverslip before the hemocytometer slide was placed under the microscope. Cells were visualized and counted under the microscope. For each sample at least 500 cells were counted, and the percent of dead to live cells was calculated. The dead cells take up the dye and appear blue, while the live cells exclude dye and appear translucent. The percentage of cell viability was calculated using Equations (5) and (6):

(5) $$\%\;\mathrm{Cytotoxicity}=\frac{T_{dead}-C_{dead}}{T_{total}}\times 100\%$$

% Viability = 100 − % Cytotoxicity(6)

where T*_dead_* is the number of dead cells in the treated tube, C*_dead_* is the number of dead cells in the control tube, and T*_total_* is the total number of cells (dead + live) in the treated tube.

#### 3.6.3. Detection of DNA Fragmentation

The cells were treated with the Ru(III) complex (**2**) (IC_50_ = 117.6 µg/mL) for 4 h. MCF7 and Caco2 cells were collected and genomic DNA was extracted using a Gene JET genomic DNA extraction kit following the manufacturer’s protocol (Thermo Scientific, #K0721). The extracted DNA samples were run on a 1.5% agarose gel at 50 V for 2 h and the fragment pattern was visualized by ethidium bromide staining under a UV transilluminator.

#### 3.6.4. Cell-Cycle Analysis Using Flow Cytometry

The Ru(III) complex (**2**) (117.6 μg/mL) was added to MCF7 and Caco2 cells at a confluence of 70–80% and incubated for 24 h (in a CO_2_ incubator). The cells were then digested with warm trypsin-EDTA + warm PBS-EDTA (0.25%) (500 + 500 μL) with incubation for 10 min at 37 °C. The mixture was centrifuged (450 rpm) for 5 min, then the supernatant was removed. The mixture was washed twice in warm PBS and the cell pellet was re-suspended in 500 µl warm PBS, centrifuged and the supernatant decanted. A volume of 150 μL PBS + 350 μL ice-cold 70% ethanol was added and incubated at 4 °C for 1 h to fix the cells. To remove ethanol, the mixture was centrifuged at 350 rpm for 10 min and then the supernatant was removed. The mixture was washed twice in warm PBS and the cells were re-suspended in 500 μL of warm PBS, centrifuged, and the supernatant was removed. The cells were resuspended in 100 μL PBS and stored at 4 °C for up to four days. In darkness, the cells were stained with 100 µL of propidium iodide (PI) solution + 50 µL RNase A solution (100 µg/mL) and incubated in darkness for 30–60 min. The stained cells were read in an Attune flow cytometer (Applied Bio-system, Foster City, CA, USA).

### 3.7. In Vivo Assays

#### 3.7.1. Animal Experiments

Female albino mice (seven weeks old, 23–25 g), bread in the Department of Chemistry, Faculty of Science, Kafrelsheikh University animal house, were fed a standard pellet diet and had free access to water. They were housed in polypropylene cages at 22 °C with a 12 h light and dark cycle. The animal procedures were approved and maintained in accordance with the guidelines of the Animal Ethical Committee of Kafrelsheikh University, Egypt.

#### 3.7.2. Median Lethal Dose (LD_50_) Determination

The LD_50_ was estimated by the method of Enegide [108]. Briefly, different concentrations of compound (**2**) was prepared by dissolving a certain weight in 2% DMSO and completed with sterile saline then vortexed and left for 5 min until the solution was clear. Three hundred µL of each concentration (10, 20, 40, 60, 80, 100, 120, and 140 mg/kg) was injected intraperitoneally to eight groups of mice (each one containing four mice), then mortality was recorded after 24 h.

#### 3.7.3. In Vivo Tumor Cell Transplantation

The mice were divided into the following eight groups (10 mice in each group):

- Control group: Mice were intraperitoneally injected with a vehicle (2% DMSO + sterile saline) for 12 days.
- **I**, **II,** and **III** control groups: Healthy normal mice were intraperitoneally injected with 6, 12, and 18 mg/kg of complex (**2**), respectively, for 12 days.
- EAC group: Mice were intraperitoneally injected by a single dose of 2.5 × 10^6^ viable Ehrlich ascites carcinoma (EAC) cells in PBS. In brief, EAC cells were withdrawn from the EAC mouse’s intraperitoneal cavity, washed, and centrifuged three times with sterile saline. Then, the pellets were suspended in sterile PBS and counted by the trypan blue exclusion assay, which confirmed that the viability of the cells was 98%.
- **I**, **II,** and **III** treated groups: Firstly, 30 healthy mice were intraperitoneally injected with a single dose of 2.5 × 10^6^ viable EAC cells. After one day; the mice were divided into I, II, and III treated groups (10 mice each group), intraperitoneally injected with 6, 12, and 18 mg/kg Ru(III) complex, respectively, for 12 days.

The animals were monitored for EAC growth by measuring abdominal size. At the end of the experiment all animals were fasted overnight then sacrificed. Serum samples were collected to study the biological parameters such as liver and kidney function as well as antioxidant enzyme activity. Liver samples from each group were frozen immediately in liquid nitrogen for molecular analysis.

#### 3.7.4. Biochemical Parameters

*Liver function tests.* Serum GPT and GOT activities were measured by the method of Murray [109,110] with a standard kit. The enzyme activities were estimated by measuring the decrease in absorbance of NADH at 340 nm. Bilirubin concentration was estimated by the colorimetric method of Kaplan et al. [111]. Albumin concentration was measured colorimetrically by the color change of bromocresol from yellow-green to green-blue [112].

*Kidney function tests*. Creatinine was estimated colorimetrically by reacting with alkaline picrate forming a red complex [112]. Urea was hydrolyzed enzymatically into ammonia which reacts with several compounds to form a green complex that can be measured colorimetrically [113]. Uric acid was estimated by the enzymatic method of Schultz [112].

#### 3.7.5. Oxidative Stress Markers

The SOD activity in serum samples was evaluated as described by Dechatelet et al. [114]. GSH was assayed in serum samples by detection of a yellow colored product following a reaction with 5,5’-dithiobis(2-nitrobenzoic acid) (DTNB) [115]. The catalase activity was estimated according to Aebi [116]. Total antioxidant capacity (TAC) was determined by the method of Koracevic et al. [117].

#### 3.7.6. Molecular Analysis by Real-Time PCR

RNA was isolated from liver tissues using a RNeasy Mini kit. The integrity and purity of RNA were assessed by 1% agarose gel electrophoresis and nanodrop, respectively. The obtained RNA was reverse transcribed to yield cDNA using Quantiscript reverse transcriptase. Specific primers for *Bax*, caspase 3, and *Bcl2* genes in addition to the housekeeping gene, *β actin* were designed by the Primer 3 web-based tool based on the published mice sequences (Table 5). Quantitative real-time PCR (qPCR) was performed using QuantiTect SYBR Green qPCR Master Mix in a StepOnePlus real-time PCR system and the melting curve condition and fold change calculation were done as previously described [118].

### 3.8. Statistical Analysis

One-way ANOVA using GraphPad Prism 7 was used to determine the difference between groups. A comparison of means was carried out with Tukey’s Honestly Significant Difference test. Data were presented as a mean ± standard error of the mean (SEM) and significance was declared at *p* < 0.05.

## 4. Conclusions

We describe the synthesis and characterization of two new ruthenium(II/III) DMSO complexes with 2-aminophenyl benzimidazole (Hapbim) ligands. Through UV-Vis and fluorescence studies it was determined that both of these compounds interact with DNA via intercalation. Furthermore, in vitro cytotoxicity was evaluated against human colorectal (Caco2), breast (MCF7) cancer cells, and liver (THLE-2) normal cells, demonstrating that the complexes have moderate in vitro activity against cancer cells, but show selectivity towards the cancerous cell lines. The Ru(III) complex (**2**) showed the highest activity, possibly indicating the importance of the redox-active metal center, and also had the lowest toxicity towards the normal cells. Additional in vitro studies of (**2**) demonstrated that the complex causes cell-cycle arrest at the G2/M phase with induction of apoptosis, with an apoptosis mechanism also shown by DNA fragmentation. Significantly, compound (**2**) shows more potent anticancer activity in vivo, by removing the cancer burden via apoptosis along with increased *Bax* and caspase *3* and decreased *Bcl2* expression. The latter has been identified previously as an approach to targeting melanoma cells, for example [119]. Furthermore, (**2**) decreases oxidative stress and increases the levels of antioxidant enzymes, especially SOD, to enhance normal cell repair. Overall, compound (**2**) shows potential as a chemotherapeutic candidate, with promising activity and low levels of side effects.

The activity of compound (**2**) is also interesting in the wider context of the development of Ru anticancer compounds. As described herein, the in vitro activity and selectivity of the complex is quite modest. The complex exhibits IC_50_ values, derived from MTT cytotoxicity tests against two commonly studied cancer cell lines, that are relatively high when compared to more conventional chemotherapeutic candidates. Furthermore, the therapeutic index of the compound, in comparison to a normal liver cell line, is reasonable but lower than cisplatin in the same studies. These observations initially suggest that complexes (**1**) and (**2**) are not strong candidates as potential drugs. However, the in vivo studies tell a very different story. As we show here, compound (**2**) is effective in treating EAC in a mouse model, and shows low off-target toxicity, indicating that compounds of this type could be effective chemotherapeutics with low levels of side effects. These observations emphasize that standard in vitro studies may not be sufficient to identify promising ruthenium anticancer candidates [22,70] and it is possible that potentially active ruthenium complexes may have been overlooked in previous studies for this reason.

## Figures and Tables

**Table 1:** Interaction study of complexes (**1**) and (**2**) with EB-DNA.

Complex	K_q_ (M^−1^)	K_app_ (M^−1^)
(**1**)	1.96 ± 0.04 × 10^3^	1.08 ± 0.11 × 10^7^
(**2**)	8.64 ± 0.15 × 10^3^	1.72 ± 0.28 × 10^7^

**Table 2:** In vitro cytotoxicity given as IC_50_ in µg/mL and µM of Hapbim and complexes (**1**), (**2**), and cisplatin against human breast cancer (MCF-7), human colorectal cells lines (Caco2), and normal liver cell line (THLE-2).

Compound	MCF-7		Caco2		THLE-2	
	µg/mL	µM	µg/mL	µM	µg/mL	µM
**Hapbim**	61 ± 4	290 ± 20	80 ± 5	380 ± 20	1140 ± 80	5500 ± 100
**(1)**	170 ± 10	320 ± 20	155 ± 9	290 ± 20	990 ± 70	1800 ± 100
**(2)**	118 ± 8	230 ± 10	129 ± 8	250 ± 10	1280 ± 80	2500 ± 100
**Cisplatin**	22 ± 1	73 ± 5	18 ± 1	60 ± 5	650 ± 60	2200 ± 100

**Table 3:** In vitro therapeutic indices (IC_50_ of normal cell/IC_50_ of cancer cell line) of Hapbim and complexes (**1**), (**2**), and cisplatin in MCF-7 and Caco2 human cancer cells lines vs. THLE-2 normal cells.

Therapeutic Index	Hapbim	(1)	(2)	Cisplatin
**IC_50_ of THLE-2 cell/IC_50_ of MCF-7**	18.5	5.8	10.89	29.3
**IC_50_ of THLE-2 cell/IC_50_ of** **Caco2**	14.2	6.4	9.95	35.9

**Table 4:** Mean serum levels of liver and kidney function tests. Compound (**2**) dose concentrations: **I** = 6 mg/kg, **II** = 12 mg/kg, and **III** = 18 mg/kg.

Group	GOT (U/L)	GPT (U/L)	Albumin (g/dL)	Bilirubin (mg/dL)	Creatinine (mg/dL)	Urea (mg/dL)	Uric Acid (mg/dL)
**Control**	36.1 ± 2.4	16.7 ± 0.6	2.9 ± 0.03	0.13 ± 0.02	0.4 ± 0.03	35.4 ± 0.5	2.85 ± 0.04
**I control**	41.8 ± 2.9 ^b^	15.6 ± 1.6 ^b^	2.3 ± 0.1 *	0.15 ±0.02 ^b^	0.37 ±0.1 ^b^	33.5 ± 0.7 ^b^	2.9 ± 0.1 ^b^
**II control**	54.9 ± 2.2 *	17.2 ± 0.5 ^b^	2.7 ± 0.1 ^b^	0.13 ± 0.02 ^b^	0.7 ± 0.02 *	31.7 ± 1.2 ^b^	2.97 ± 0.1 ^b^
**III control**	68.8 ± 3.6 *	19.6 ± 0.3 *	2.3 ± 0.1 *	0.26 ±0.02 *	0.75±0.02 *	36.7 ± 1.6 ^b^	2.77 ± 0.1 ^b^
**EAC**	250 ± 42.1 *	93.7 ± 4.8 *	0.5 ± 0.1 *	0.3 ± 0.03 *	1.1 ± 0.1 *	41.5 ±0.5 *	3.74 ± 0.04 *
**I treated**	248 ± 15.6 ^a^	15.2 ± 0.7 ^#^	0.9 ± 0.03 ^#^	0.18 ± 0.03 ^#^	0.7 ± 0.01 ^#^	34.5 ± 0.4 ^#^	2.6 ± 0.1 ^#^
**II treated**	179 ± 10.8 ^#^	17.8 ± 0.6 ^#^	2.7 ± 0.3 ^#^	0.2 ± 0.03 ^#^	0.7 ± 0.1 ^#^	34.2 ± 0.6 ^#^	2.7 ± 0.1 ^#^
**III treated**	145 ± 39.3 ^#^	31.9 ± 7.8 ^#^	2.6 ± 0.1 ^#^	0.18 ±0.02 ^#^	1 ± 0.1 ^a^	33.3 ± 1.2 *^#^*	2.8 ± 0.1 *^#^*

Values represent mean ± SEM. * *p* < 0.05, compared to the control group, ^#^ *p* < 0.05, compared to the EAC group. ^a^ Non-significant *p* > 0.05, compared to the EAC group. ^b^ Non-significant *p* > 0.05, compared to the control group.

**Table 5:** Primers used for real-time PCR.

Gene	Forward Primer	Reverse Primer
*Bax*	ACACCTGAGCTGACCTTG	AGCCCATGATGGTTCTGATC
*Bcl2*	AGTACCTGAACCGGCATCTG	CATGCTGGGGCCATATAGTT
*Caspase 3*	TTAATAAAGGTATCCATGGAGAACACT	TTAGTGATAAAAATAGAGTTCTTTTGTGAG
*β actin*	AAGTCCCTCACCCTCCCAAAAG	AAGCAATGCTGTCACCTTCCC

## Supplementary Materials

The following are available online: Figure S1: IR spectra; Figure S2: NMR spectra; Figure S3: Mass spectra; Figures S4 and S5: UV-Visible spectra; Figures S6 and S7: Cytotoxicity data; Figures S8–S10 and Tables S1–S6: DFT calculation.
