# Supplementary material for: Ruthenium(II)/(III) DMSO-Based Complexes of 2-Aminophenyl Benzimidazole with In Vitro and In Vivo Anticancer Activity
Source: Molecules. 2020 Sep 18;25(18):4284. doi: 10.3390/molecules25184284 (PMC7570852; doi:10.3390/molecules25184284)
Supplement: Supplementary file 1 [file molecules-25-04284-s001.pdf]

Ruthenium(II)/(III) DMSO-Based Complexes of 2-Aminophenyl Benzimidazole with In Vitro and In Vivo Anticancer Activity

Shadia A. Elsayed [1,*], Shane Harrypersad [2], Heba A. Sahyon [3,*], Mohammed Abu El-Magd [4] and Charles J. Walsby [2,*]

[1] Chemistry Department, Faculty of Science, Damietta University, New Damietta 34517, Egypt; shadia.elsayed@du.edu.eg
[2] Department of Chemistry, Simon Fraser University, 8888 University D, Burnaby, British Columbia, V5A 1S6, Canada; cwalsby@sfu.ca
[3] Chemistry Department, Faculty of Science, Kafrelsheikh University, Kafrelsheikh, 33516, Egypt; heba_sahuon@sci.kfs.edu.eg
[4] Anatomy and Embryology Department, Faculty of Veterinary Medicine, Kafrelsheikh University, Egypt; mohamed.abouelmagd@vet.kfs.edu.eg

* Correspondence: shadia.elsayed@du.edu.eg (S.A.E.); heba_sahuon@sci.kfs.edu.eg (H.A.S.); cwalsby@sfu.ca (C.J.W.)

Supporting Information

Table of Contents

1) FTIR spectra ……………………………………………..……………………………..…S4

- **Fig. S1.** FTIR spectra of Fig. S1: IR spectra of **(a)** 2-aminophenyl benzimidazole ligand (Hapbim), **(b)** [Ru(II)$Cl_2$$(DMSO)_2$(Hapbim)] (**1**) and **(c)** [Ru(III)Cl_3(DMSO)(Hapbim)] (**2**)

2) NMR spectra ……………………………………..………………..…..…………. S5-S8

- **Fig. S2a.** Full ^{1}H NMR spectrum of 2-aminophenyl benzimidazole ligand (Hapbim) in DMSO-d_6.
- **Fig. S2b.** Expanded part of ^{1}H NMR spectrum of 2-aminophenyl benzimidazole ligand (Hapbim).
- **Fig. S2c.** ^{1}H NMR spectrum of [Ru(II)$Cl_2$$(DMSO)_2$(Hapbim)] (**1**) in DMSO-d_6.
- **Fig. S2d.** Expanded part of ^{1}H NMR spectrum of [Ru(II)$Cl_2$$(DMSO)_2$(Hapbim)] (**1**) in DMSO-d_6.
- **Fig. S2e.** Paramagnetic ^{1}H NMR spectrum of [Ru(III)Cl_3(DMSO)(Hapbim)] (**2**) in DMSO-d_6.
- **Fig.S2f**. ^{13}C NMR spectrum of [Ru(II)$Cl_2$$(DMSO)_2$(Hapbim)] (**1**) in DMSO-d_6.
- **Fig.S2g.** ^{13}C NMR spectrum of [Ru(III)Cl_3(DMSO)(Hapbim)] (**2**) in DMSO-d_6

3) Mass spectra ……………………………………………………………………….…...S9-S10

- **Fig. S3a.** HRES-MS spectrum of [Ru(II)Cl_2($(DMSO)_2$(Hapbim)] (**1**)
- **Fig. S3b.** HRES-MS spectrum of [RuCl_3(Hapbim)(DMSO)] (**2**)

4) UV-visible spectra ………………………………………………………………..S11-S13

- **Fig. S4.** UV-visible spectra of Hapbim (3.3 × 10^{-5} M), [Ru(II)$Cl_2$$(DMSO)_2$(Hapbim)] (**1**) (4×$10^{-5}$ M) and [Ru(III)Cl_3(DMSO)(Hapbim)] (**2**) (4 × 10^{-5} M) in DMSO.
- **Fig. S5a.** Time-dependent electronic spectra of 4 × 10^{-5} M of u(II)$Cl_2$$(DMSO)_2$(Hapbim)] (**1**) in 96 %PBS, 4% DMSO.
- **Fig. S5b.** Time-dependent electronic spectra of 4 × 10^{-5} M of [Ru(II)$Cl_2$$(DMSO)_2$(Hapbim)] (**1**) in PBS (a) double chloride concentration, (b) double DMSO concentration.
- **Fig. S5c.** Time-dependent electronic spectra of 4 × 10^{-5} M of [Ru(III)Cl_3(DMSO)(Hapbim)] (**2**) in 96 %PBS, 4% DMSO.
- **Fig. S5d.** Time-dependent electronic spectra of 4 × 10^{-5} M of [Ru(III)Cl_3(DMSO)(Hapbim)] (**2**) in PBS (a) double chloride concentration, (b) double DMSO concentration.

5) Cytotoxicity ……………….……………………………………......……………S14- S15

- **Fig. S6.** Cytotoxic effect of the isolated compounds on MCF7, Caco2 and THLE-2 cells determined by MTT assay. Results are expressed as percentage of cell viability relative to vehicle- treated control cells.
- **Fig. S7.** Cytotoxic effect of complex [Ru(III)Cl_3(DMSO)(Hapbim)] (**2**) on EAC cells

6) DFT Calculations……….……………………………………......……………S16- S24

- **Fig S8.** DFT calculated structure of Hapbim ligand
- **Table S1.** Calculated coordinates of Hapbim ligand
- **Table S2.** Calculated bond lengths of Hapbim ligand
- **Table S3.** Calculated bond angles of Hapbim ligand
- **Fig S9.** DFT calculated structure of [Ru(II)$Cl_2$$(DMSO)_2$(Hapbim)] (**1**)
- **Table S4.** Calculated coordinates of [Ru(II)$Cl_2$$(DMSO)_2$(Hapbim)] (**1**)
- **Table S5.** Calculated bond lengths of [Ru(II)$Cl_2$$(DMSO)_2$(Hapbim)] (**1**)
- **Table S6.** Calculated bond angles of [Ru(II)$Cl_2$$(DMSO)_2$(Hapbim)] (**1**)
- **Fig S10.** DFT calculated structure of [Ru(III)Cl_3(DMSO)(Hapbim)] (**2**)
- **Table S7.** Calculated coordinates of [Ru(III)Cl_3(DMSO)(Hapbim)] (**2**)

- **Table S8.** Calculated bond lengths of [Ru(III)Cl_3(DMSO)(Hapbim)] (**2**)
- **Table S9.** Calculated bond angles of of [Ru(III)Cl_3(DMSO)(Hapbim)] (**2**)
- **Fig S11.** Calculated spin-density distribution of [Ru(III)Cl_3(DMSO)(Hapbim)] (**2**), shown with isovalue of 0.005
- **Table S10.** Calculated Mulliken spin densities of [Ru(III)Cl_3(DMSO)(Hapbim)] (**2**)

1. FTIR spectra

(a)

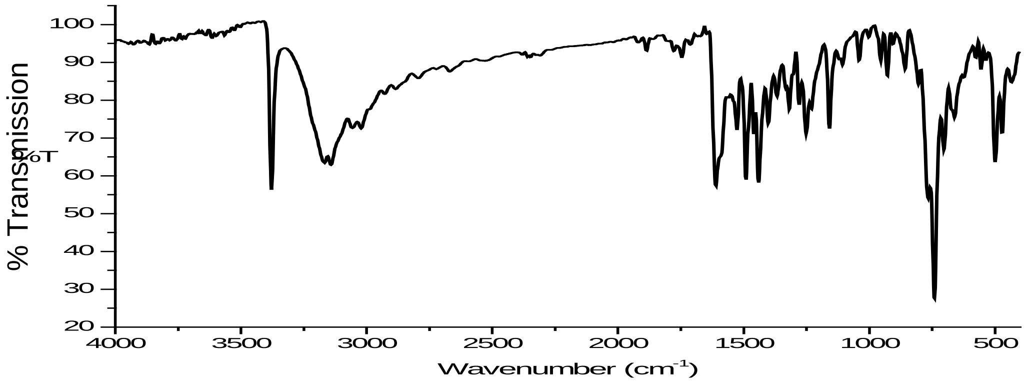

(b)

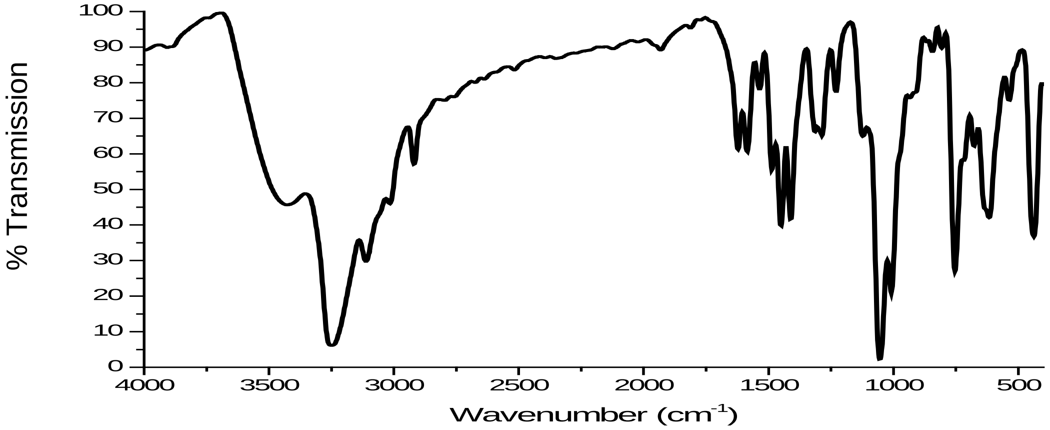

(c)

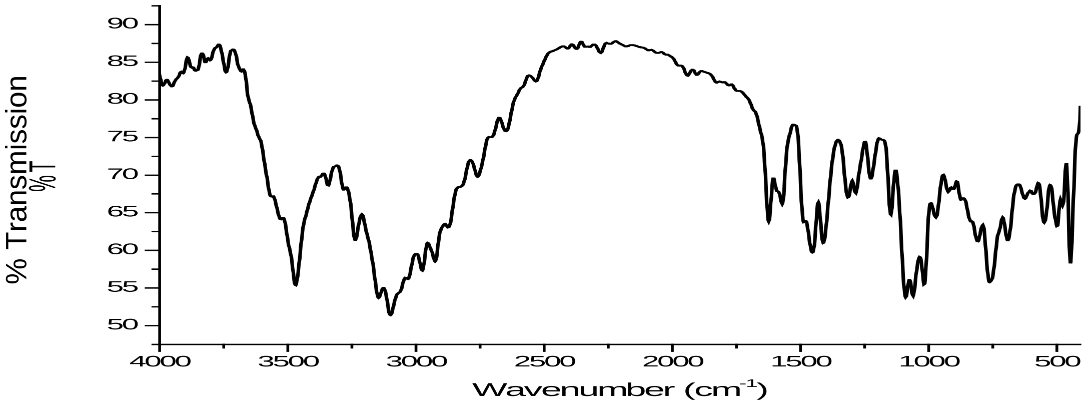

Fig. S1. IR spectra of **(a)** 2-aminophenyl benzimidazole ligand (Hapbim),
(b) [Ru(II)Cl_2(DMSO)$_2$(Hapbim)] (**1**) and **(c)** [Ru(III)Cl_3(DMSO)(Hapbim)] (**2**).

2. NMR spectra

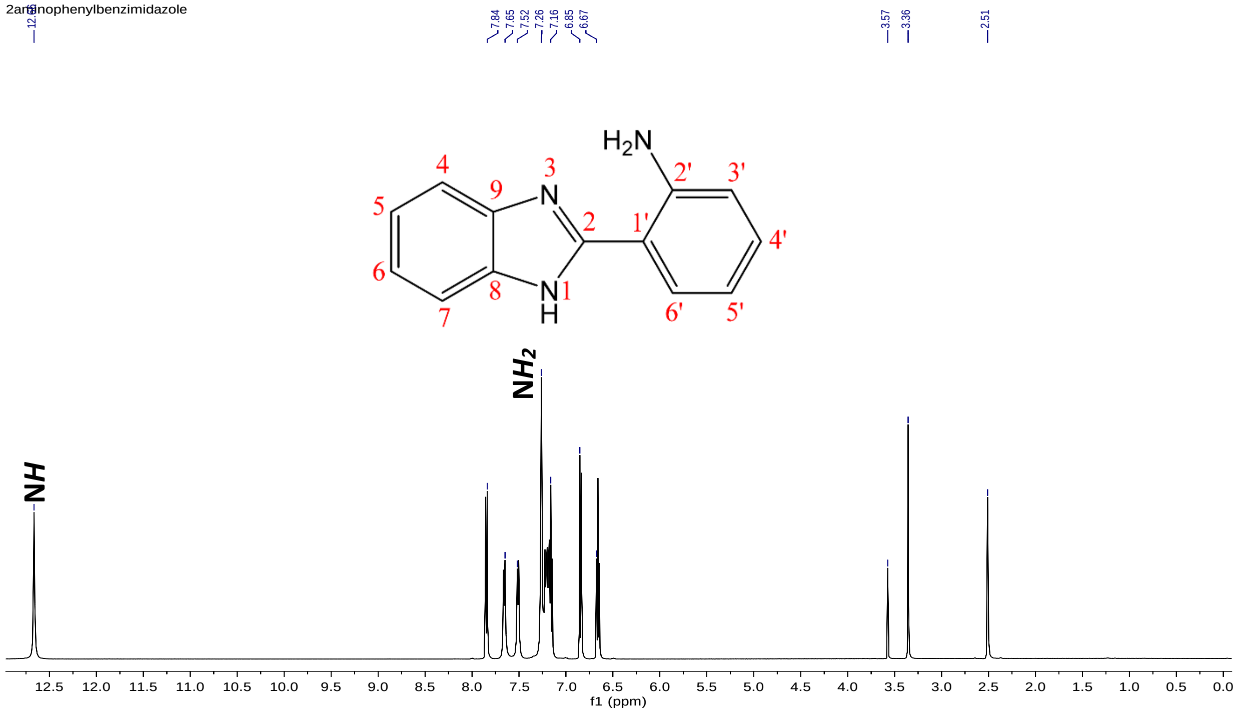

Fig. S2a. Full ^{1}H NMR spectrum of 2-aminophenyl benzimidazole ligand (Hapbim) in DMSO-d_6.

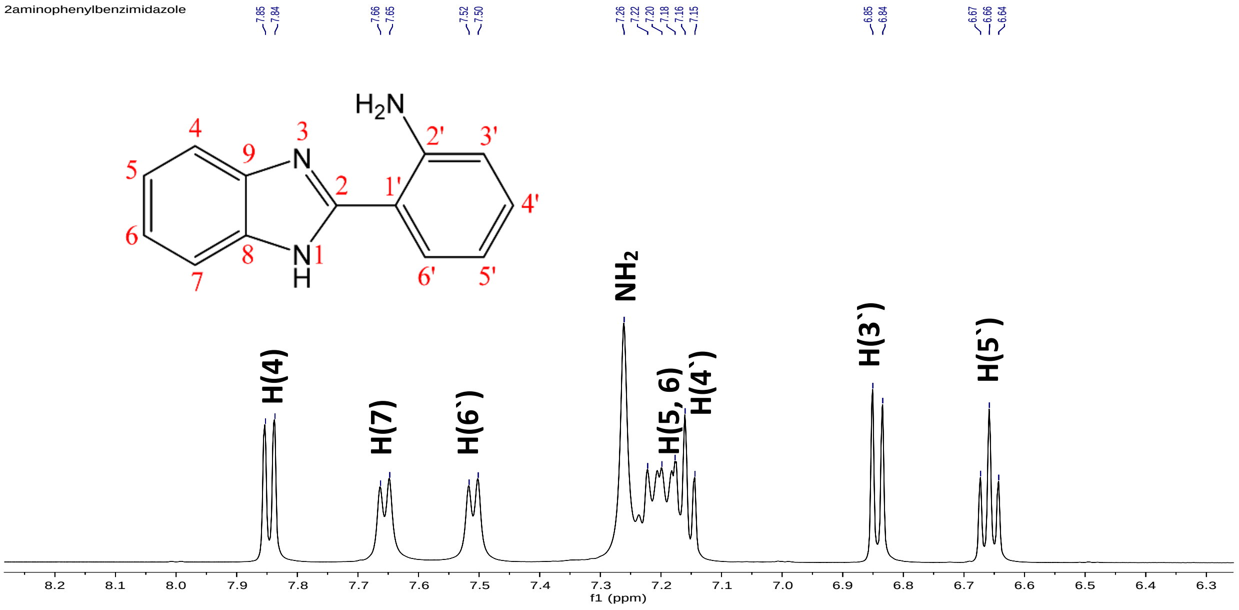

Fig. S2b. Expanded part of ^{1}H NMR spectrum of 2-aminophenyl benzimidazole ligand (Hapbim).

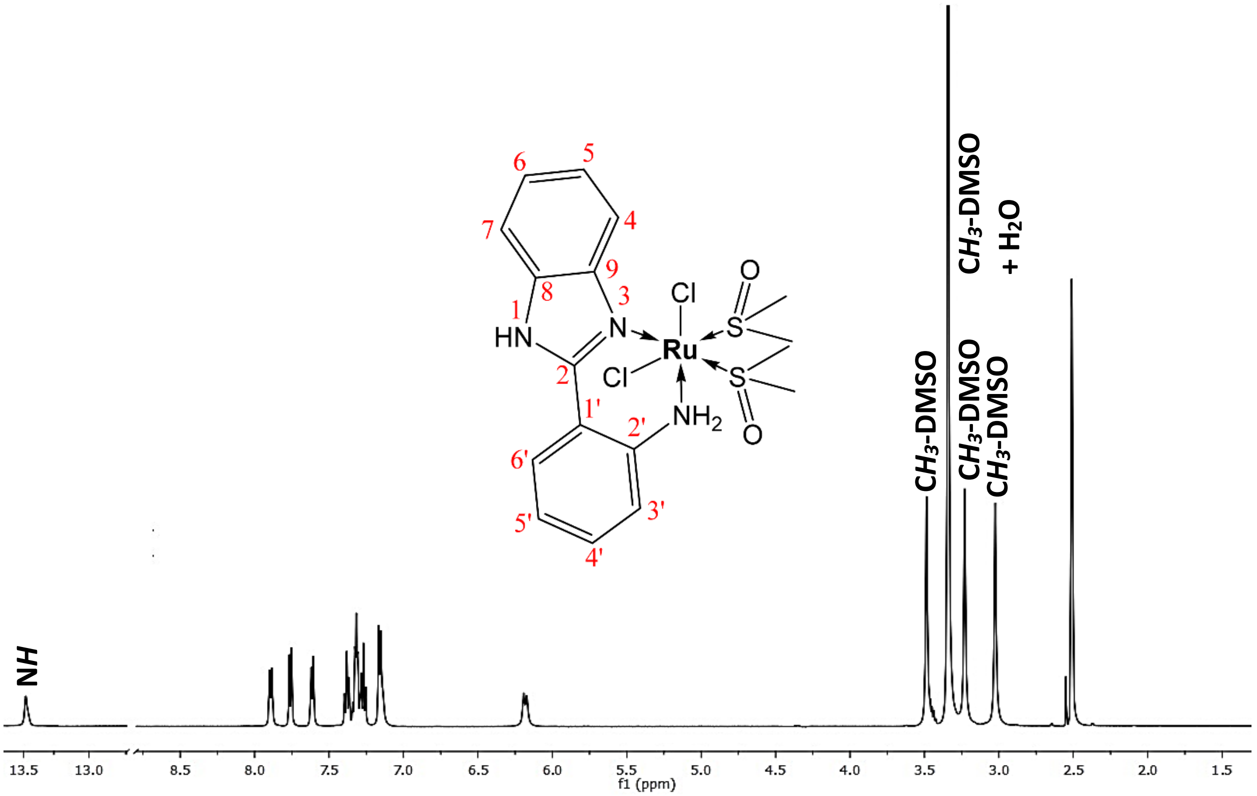

Fig. S2c. ^{1}H NMR spectrum of $[Ru(II)Cl_2(DMSO)_2(Hapbim)]$ (**1**) in DMSO-d_6.

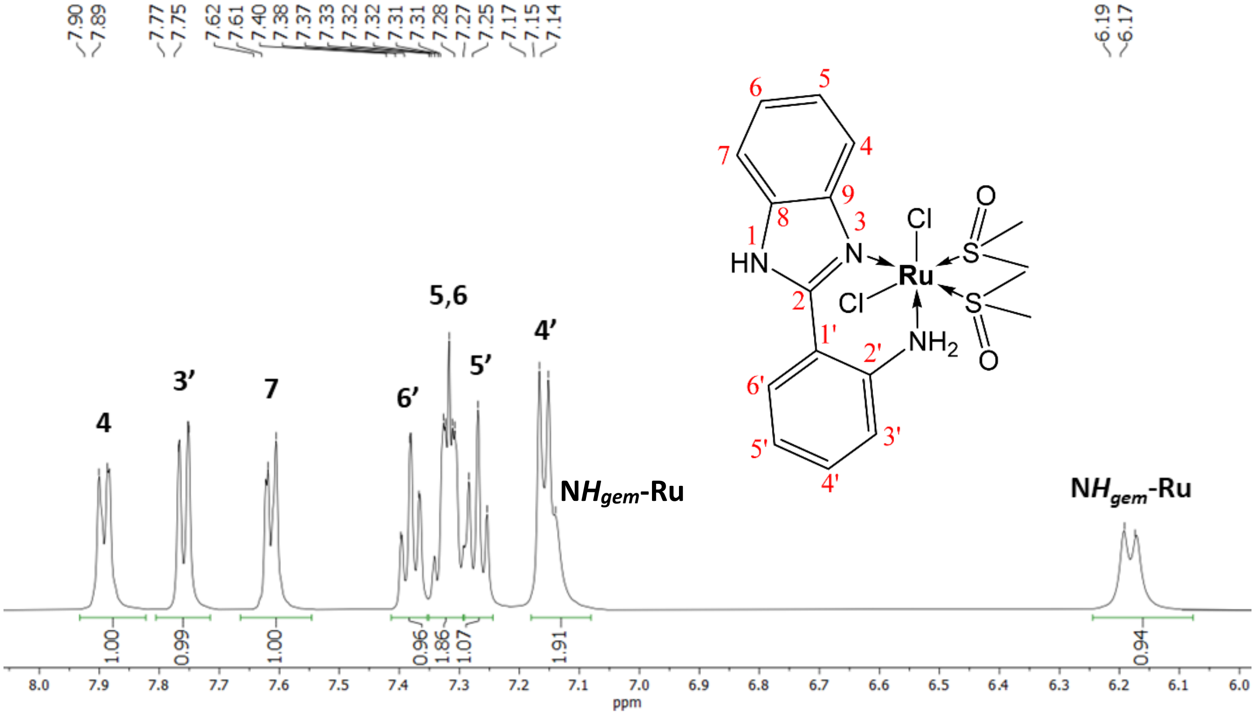

Fig. S2d. Expanded part of ^{1}H NMR spectrum of $[Ru(II)Cl_2(DMSO)_2(Hapbim)]$ (**1**) in DMSO-d_6.

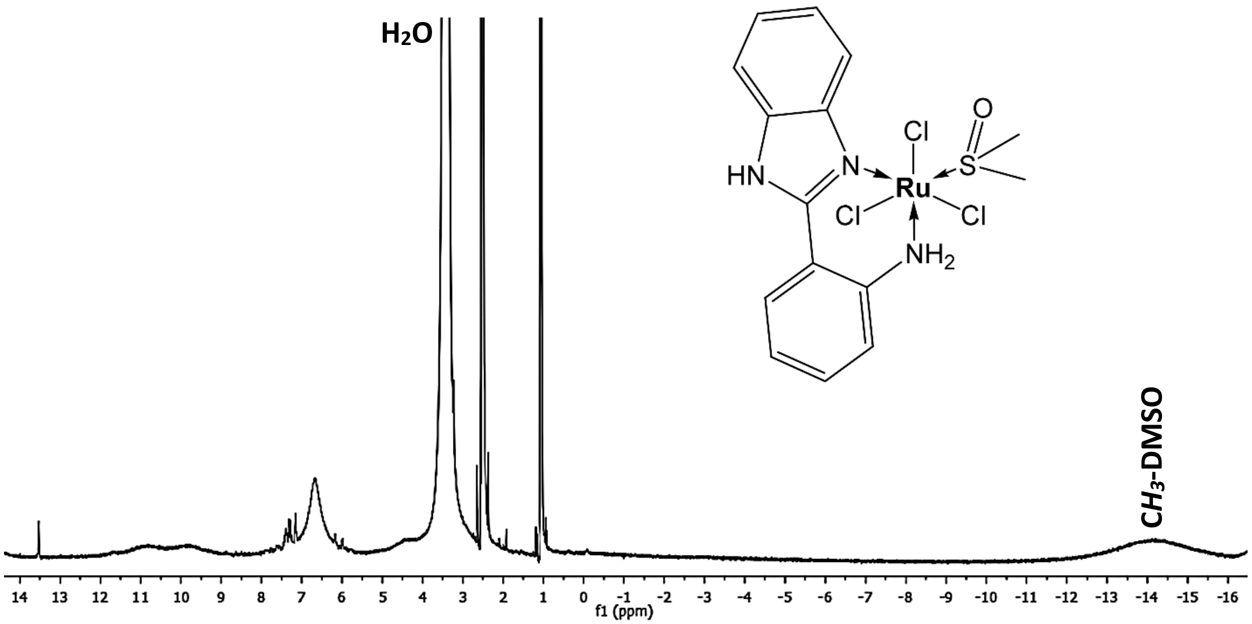

Fig. S2e. Paramagnetic ^{1}H NMR spectrum of $[Ru(III)Cl_3(DMSO)(Hapbim)]$ (**2**) in DMSO-d_6

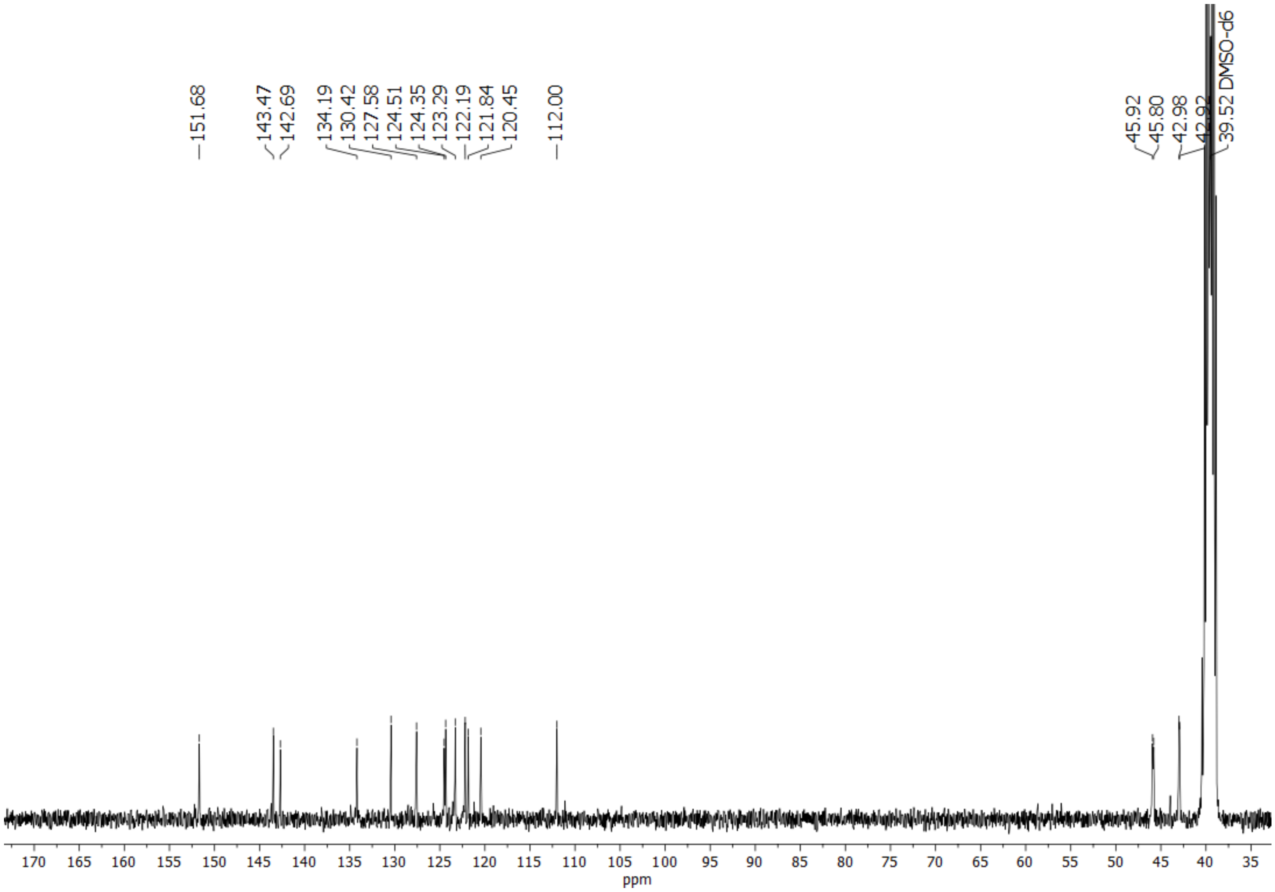

Fig.S2f. ^{13}C NMR spectrum of $[Ru(II)Cl_2(DMSO)_2(Hapbim)]$ (**1**) in DMSO-d_6

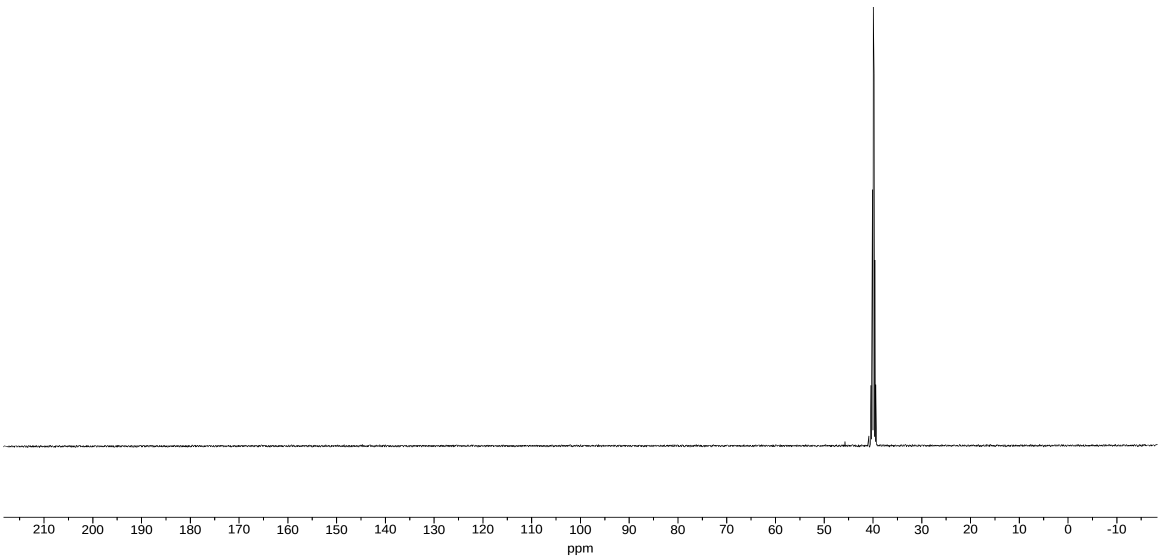

Fig.S2g. ^{13}C NMR spectrum of [Ru(III)Cl_3(DMSO)(Hapbim)] (**2**) in DMSO-d_6

3. Mass spectra

a)

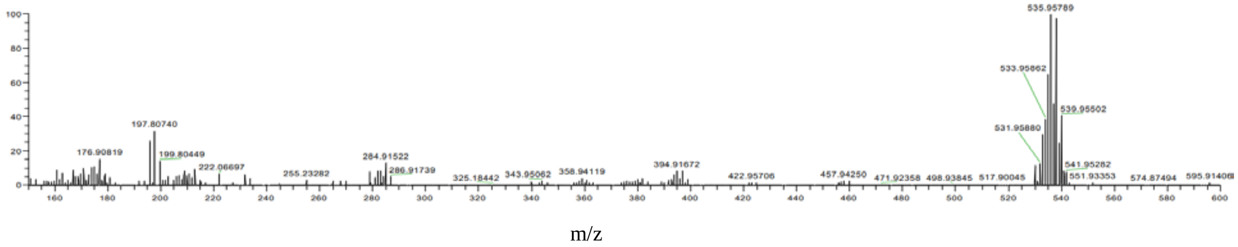

b)

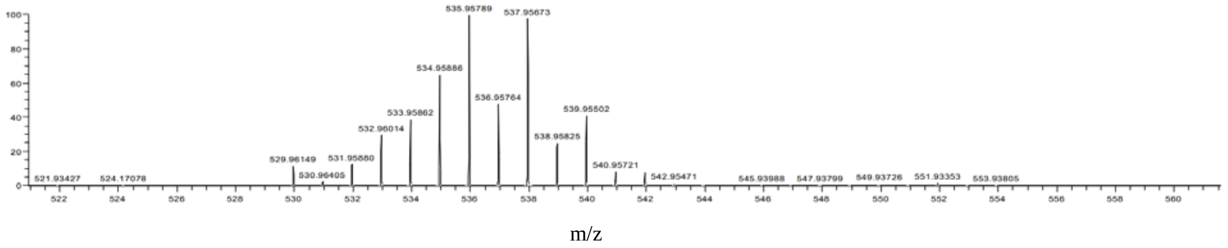

c)

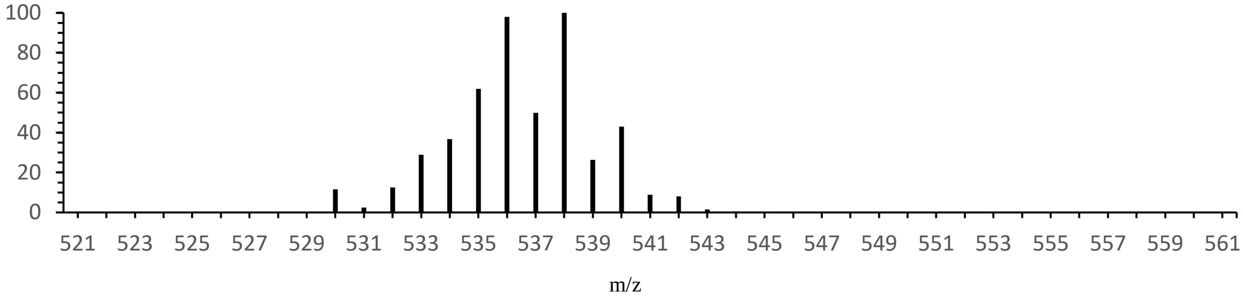

Fig. S3a. a) HRES-MS spectrum of [Ru(II)Cl_2(DMSO)$_2$(Hapbim)] (**1**), (b) expanded view of main m/z peak, (c) calculated isotopic pattern using natural abundances.

a)

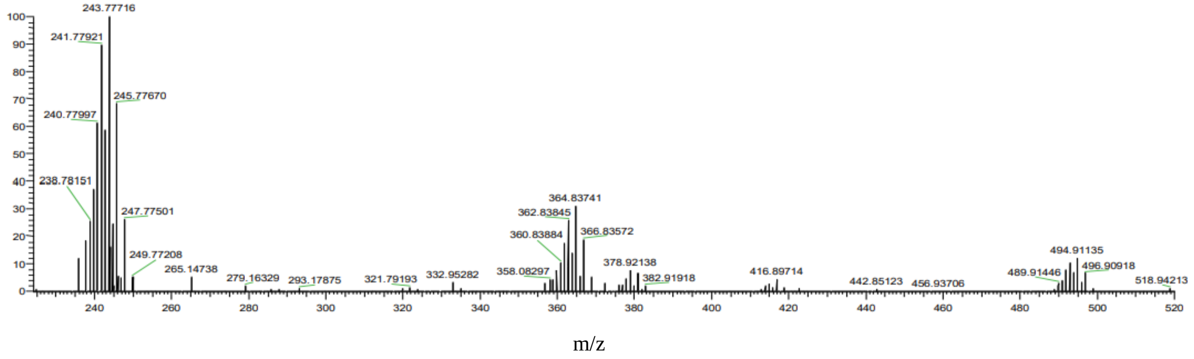

b)

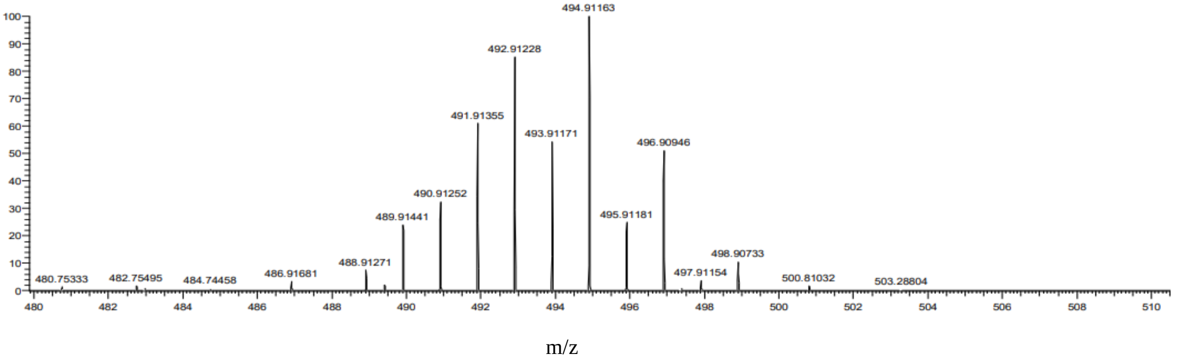

c)

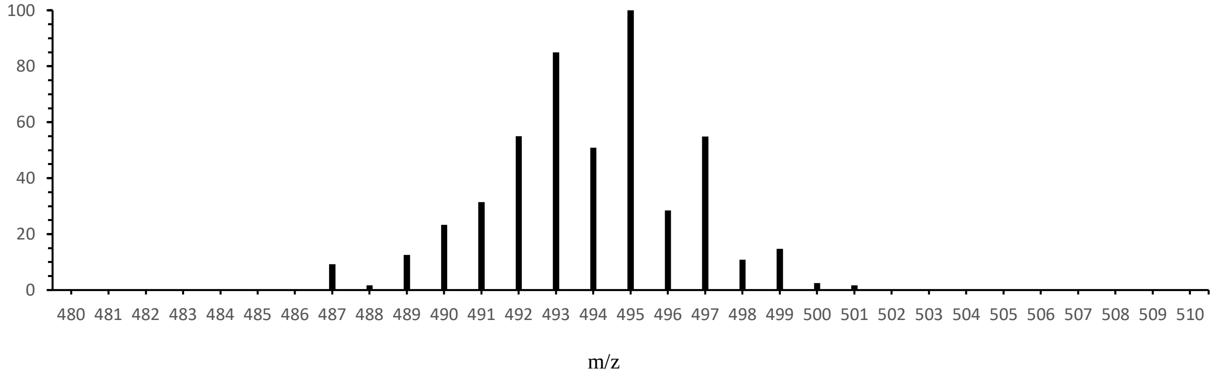

Fig. S3b. a) HRES-MS spectrum of [Ru(III)Cl_3(DMSO)(Hapbim)] (**2**), b) expanded view of main m/z peak, (c) calculated isotopic pattern using natural abundances.

4. UV-visible spectra

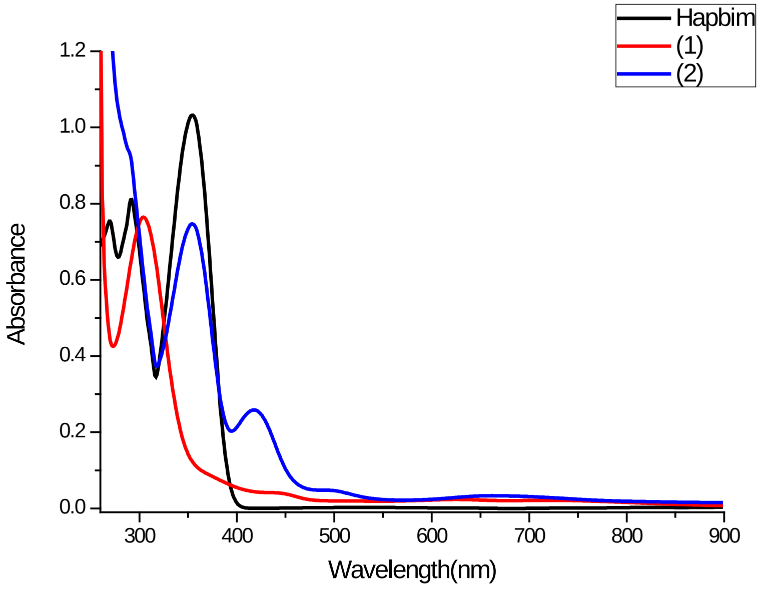

Fig. S4. UV-visible spectra of Hapbim (3.3 × 10^{-5} M), [Ru(II)Cl_2(DMSO)$_2$(Hapbim)] (**1**) (4 × 10^{-5} M) and [Ru(III)Cl_3(DMSO)(Hapbim)] (**2**) (4 × 10^{-5} M) in DMSO.

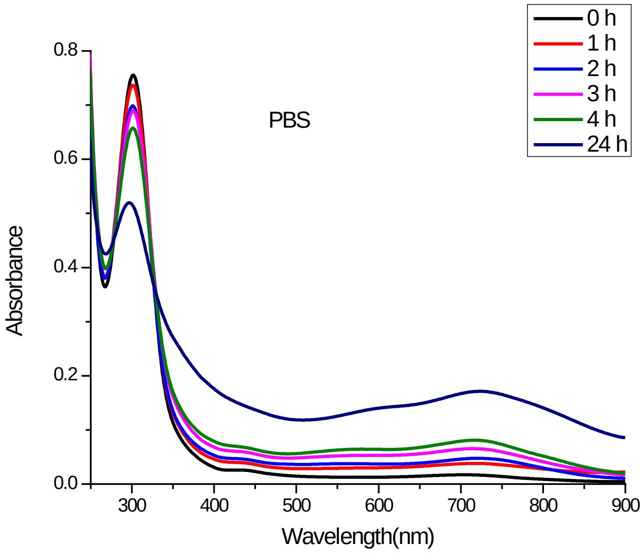

Fig. S5a. Time-dependent electronic spectra of 4 × 10^{-5} M of [Ru(II)Cl_2(DMSO)$_2$(Hapbim)] (**1**) in 96 %PBS, 4% DMSO.

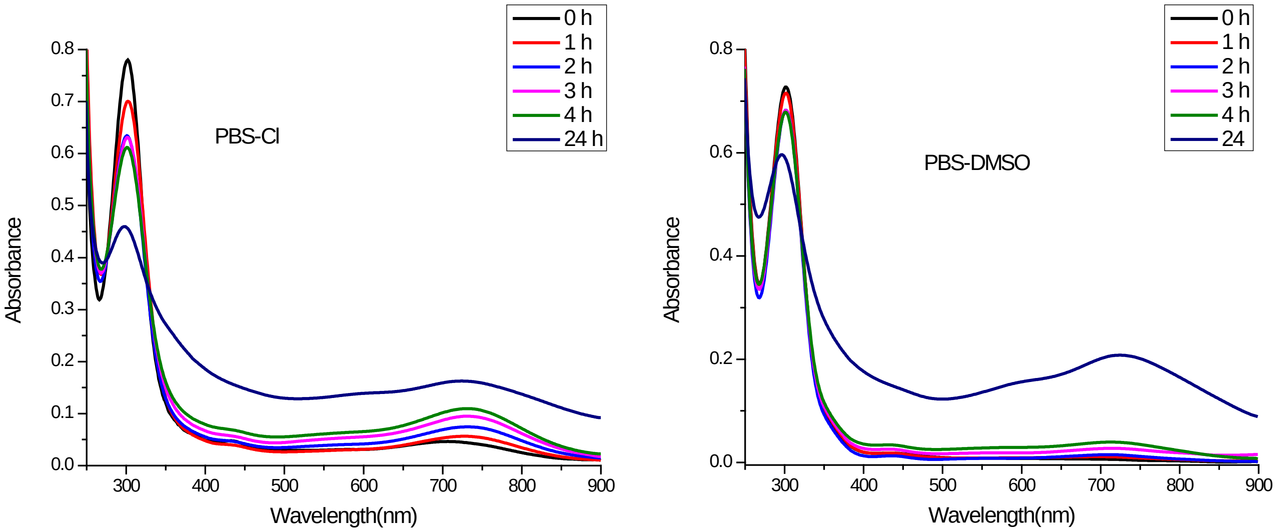

(a) **(b)**

Fig. S5b. Time-dependent electronic spectra of 4 ×10^{-5} M of [Ru(II)$Cl_2$$(DMSO)_2$(Hapbim)] (**1**) in PBS (**a**) double chloride concentration, (**b**) double DMSO concentration.

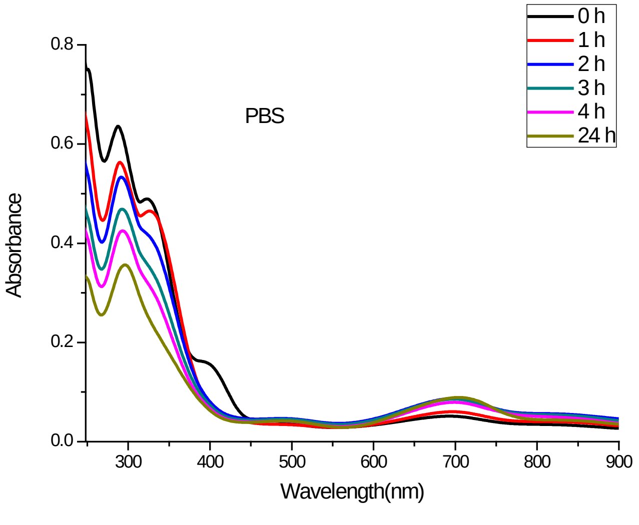

Fig. S5c. Time-dependent electronic spectra of 4 ×10^{-5} M of [Ru(III)Cl_3(DMSO)(Hapbim)] (**2**) in 96 %PBS, 4% DMSO.

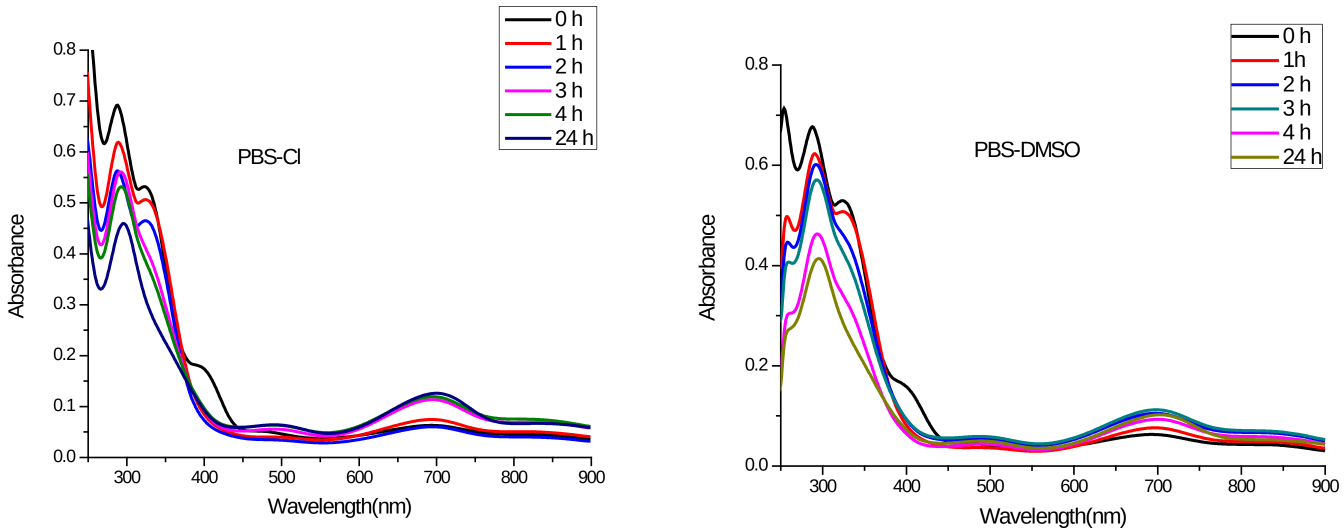

(a) **(b)**

Fig. S5d. Time-dependent electronic spectra of 4×10^{-5} M of [Ru(III)Cl_3(DMSO)(Hapbim)] (**2**) in PBS **(a)** double chloride concentration, **(b)** double DMSO concentration.

5) Cytotoxicity

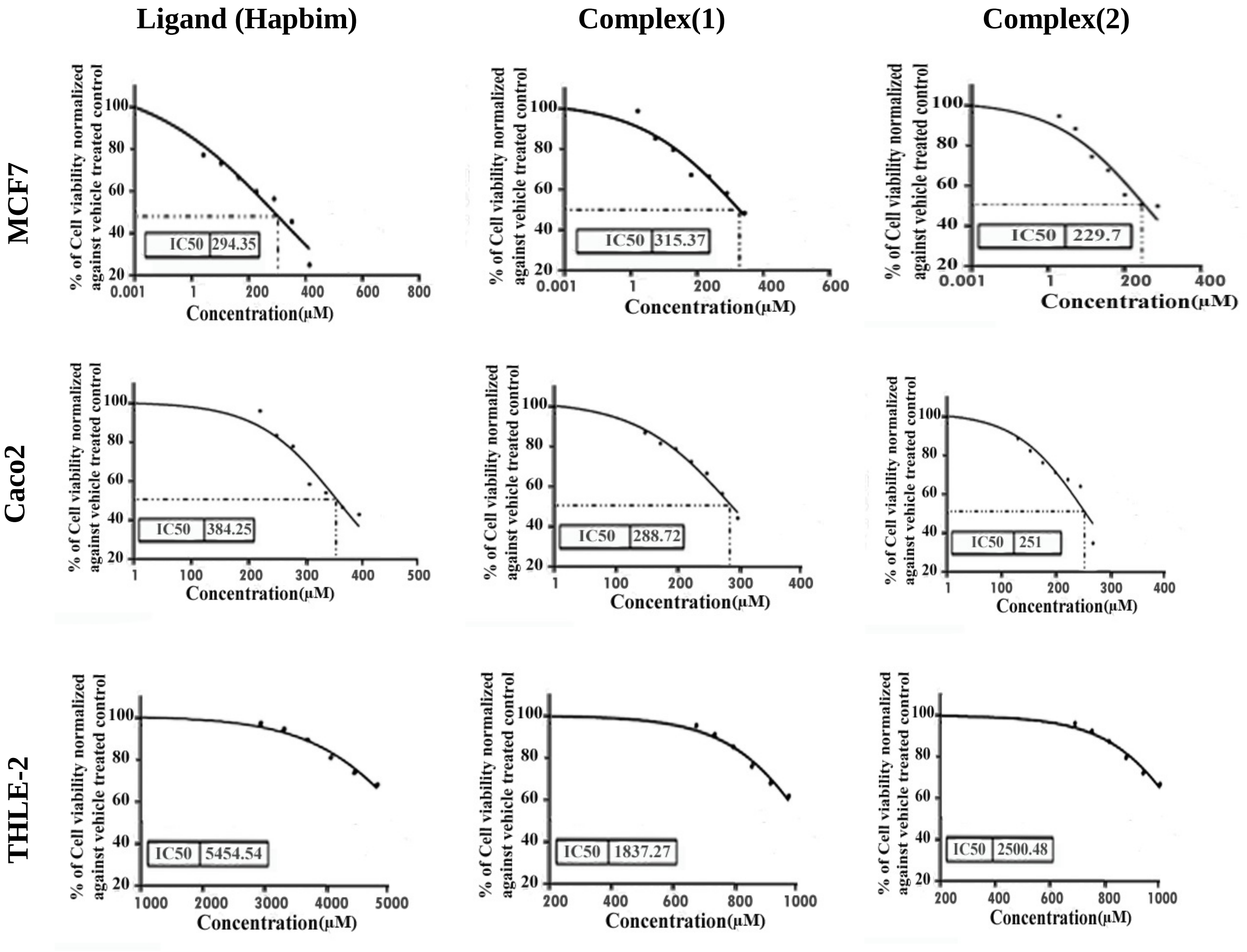

Fig. S6. Cytotoxic effect of Hapbim ligand and compounds **(1)** and **(2)** on MCF7, Caco2 and THLE-2 cells. Cells were treated with various concentrations of ligand, compound (**1**) and compound (**2**) (from 5 to 400 μM for MCF7 and Caco2 cancer cell lines) and (600 to 1000 μM for THLE-2 normal cell lines) for 24 h and the cell viability was determined by MTT assay. The results are expressed as percentage of cell viability relative to vehicle- treated control cells.

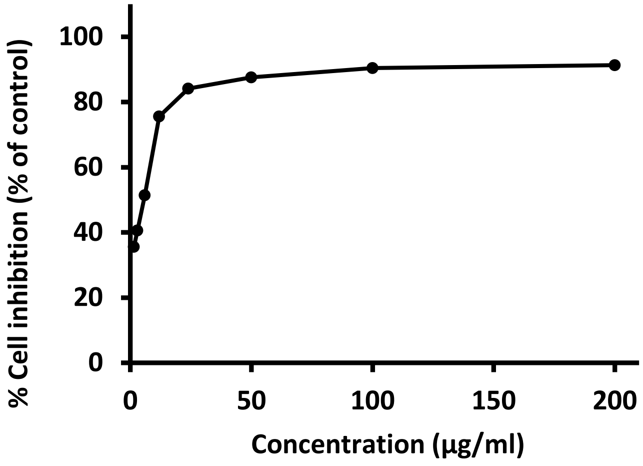

Fig. S7. Cytotoxic effect of complex [Ru(III)Cl_3(DMSO)(Hapbim)] (**2**) on EAC cells.

6) DFT Calculations

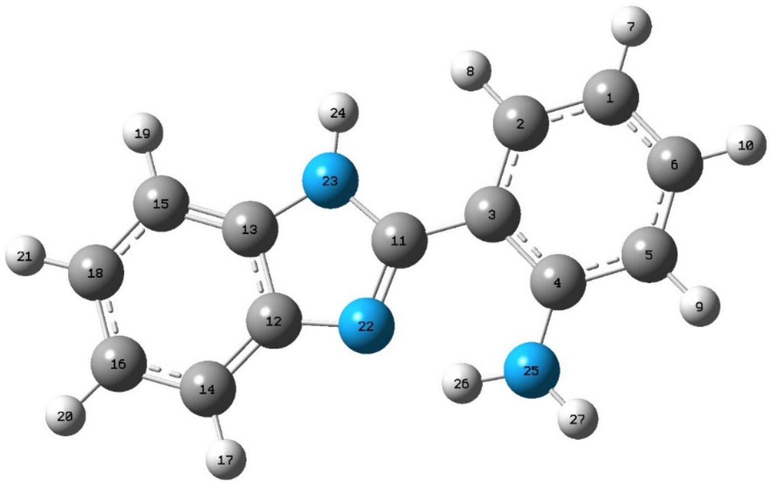

Fig S8. DFT calculated structure of Hapbim ligand

Table S1. Calculated coordinates of Hapbim ligand

Atom Label	Element	Coordinates (Å) X	Y	Z	Atom Label	Element	Coordinates (Å) X	Y	Z
1	C	3.640969	-1.56272	0.000034	15	C	-3.234032	-1.469545	0.000013
2	C	2.251669	-1.475623	0.000013	16	C	-4.30226	0.73992	0.000031
3	C	1.585463	-0.233405	0.000041	17	H	-2.99933	2.49132	0.000017
4	C	2.355426	0.974922	0.000086	18	C	-4.384074	-0.66991	0.000059
5	C	3.769102	0.868278	0.000024	19	H	-3.308169	-2.562916	-2.6E-05
6	C	4.396695	-0.372346	0.000007	20	H	-5.227511	1.327442	0.000081
7	H	4.139518	-2.537343	0.000046	21	H	-5.370189	-1.148529	0.000082
8	H	1.668115	-2.408002	0.000119	22	N	-0.57048	0.988884	0.000089
9	H	4.365217	1.790236	-0.000019	23	N	-0.69458	-1.256874	-0.00012
10	H	5.492242	-0.423026	-0.000089	24	H	-0.388859	-2.228823	-0.00036
11	C	0.149339	-0.154131	-0.000025	25	N	1.724632	2.180951	-5.5E-05
12	C	-1.907671	0.616356	-0.000044	26	H	0.668568	2.144757	-0.00017
13	C	-2.005964	-0.806812	-0.00003	27	H	2.265358	3.043826	-0.00021
14	C	-3.068322	1.398404	-0.000025					

Table S2. Calculated bond lengths of Hapbim ligand

Bond(Label-Label)		Length	Bond(Label-Label)		Length
1	2	1.392	12	14	1.3995
1	6	1.41	12	22	1.3881
1	7	1.0947	13	15	1.3955
2	3	1.4096	13	23	1.3865
2	8	1.0999	14	16	1.3986
3	4	1.4328	14	17	1.0951
3	11	1.4383	15	18	1.4007
4	5	1.4177	15	19	1.0959
4	25	1.361	16	18	1.4122
5	6	1.3903	16	20	1.096
5	9	1.0979	18	21	1.0961
6	10	1.0967	23	24	1.0189
11	22	1.3508	25	26	1.0567
11	23	1.3886	25	27	1.0183
12	13	1.4266			

Table S3. Calculated bond angles of Hapbim ligand

Angle	Angle(°)	Angle	Angle(°)	Angle	Angle(°)	Angle	Angle(°)	Angle	Angle(°)
2-1-6	118.82	3-4-25	119.88	22-11-23	110.37	13-15-18	116.84	11-23-13	108.48
2-1-7	120.68	5-4-25	121.93	13-12-14	120.02	13-15-19	122.23	11-23-14	125.11
6-1-7	120.50	4-5-6	121.15	13-12-22	109.52	18-15-19	120.93	13-23-24	126.40
1-2-3	121.79	4-5-9	118.57	14-12-22	130.46	14-16-18	121.41	4-25-26	115.65
1-2-8	118.45	6-5-9	120.28	12-13-15	122.30	14-16-20	119.50	4-25-27	120.32
3-2-8	119.75	1-6-5	120.76	12-13-23	104.99	18-16-20	119.09	26-25-27	124.04
3-3-4	119.29	1-6-10	119.76	15-13-23	132.70	15-18-16	121.49		
2-3-11	121.36	5-6-10	119.48	12-14-16	117.94	15-18-21	119.30		
4-3-11	119.35	3-11-22	125.36	12-14-17	120.36	16-18-21	119.21		
3-4-5	118.19	3-11-23	124.27	16-14-17	121.70	11-22-12	106.63		

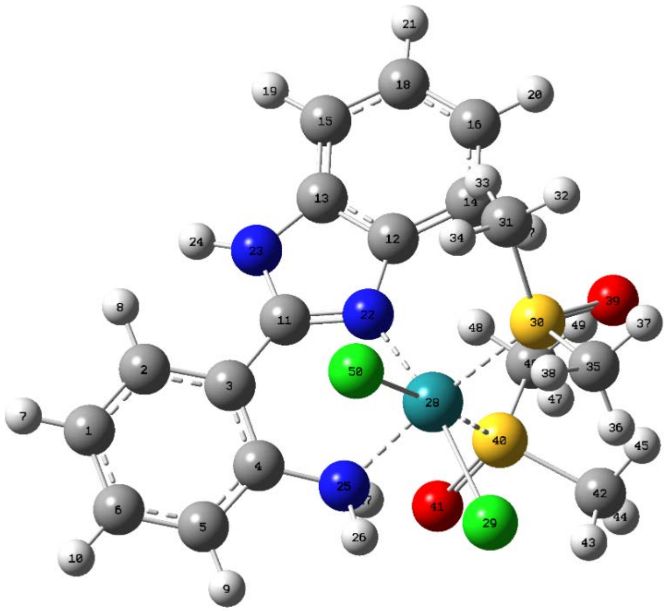

Fig S9. DFT calculated structure of [Ru(II)Cl_2(DMSO)$_2$(Hapbim)] (**1**)

Table S4. Calculated coordinates of [Ru(II)Cl_2(DMSO)$_2$(Hapbim)] (**1**)

		Coordinates (Å)					Coordinates (Å)		
**Atom Label**	**Element**	**X**	**Y**	**Z**	**Atom Label**	**Element**	**X**	**Y**	**Z**
1	C	3.577057	-1.134969	-1.325183	15	C	-2.9865	-1.307922	0.287202
2	C	2.21176	-1.063042	-0.894778	16	C	-3.960133	0.592880	1.483619
3	C	1.645363	0.174531	-0.593589	17	H	-2.728513	2.352169	1.830339
4	C	2.405508	1.352706	-0.46638	18	C	-4.069533	-0.704542	0.931852
5	C	3.738402	1.2276835	-1.164811	19	H	-3.063699	-2.308081	-1.151950
6	C	4.329476	0.042250	-1.455007	20	N	-4.833781	1.041482	1.970286
7	H	4.029301	-2.108250	-1.542300	21	H	-5.021562	-1.241966	1.004769
8	H	1.696986	-1.998531	-0.747980	22	N	-0.363425	1.181579	0.560668
9	H	4.316100	2.204375	-1.269580	23	N	-0.573807	-0.860058	-0.332500
10	H	5.375269	-0.001148	-1.778039	24	H	-0.346721	-1.695449	-0.875189
11	C	2.76453	0.198971	-0.126811	25	N	1.8237	2.63353	-0.495775
12	C	-1.672607	0.725971	0.789995	26	H	2.561129	3.341631	-0.296458
13	C	-1.799988	-0.573580	0.229427	27	H	1.208825	3.050986	-1.300753
14	C	-2.772161	1.326856	1.419773	28	Ru	0.566489	2.877936	1.120477

Table S6 continued

Atom Label	Element	Coordinates (Å) X	Y	Z	Atom Label	Element	Coordinates (Å) X	Y	Z
29	Cl	1.97778	4.816082	1.523283	40	S	-0.676504	4.133424	-0.289481
30	S	-0.682643	3.359036	2.960369	41	O	0.038497	4.038154	-1.823701
31	C	-1.011854	1.936733	4.125317	42	C	-0.822712	5.931489	0.134907
32	H	-1.469271	2.350872	5.036830	43	H	0.205188	6.279085	0.321465
33	H	-1.687991	1.266450	3.598793	44	H	-1.311980	6.437610	-0.710908
34	H	0.028431	1.470910	4.307149	45	H	-1.4267	5.956919	1.059535
35	C	0.142875	4.500914	43191623	46	C	-2.465747	3.758977	-0.600296
36	H	0.404686	5.409536	3.611014	47	H	-2.792688	4.431220	-1.408648
37	H	-0.587055	4.684521	4.975205	48	H	-2.534228	2.701013	-0.891834
38	H	1.053151	3.995976	4.531467	49	H	-2.98057	2.949627	0.356194
39	O	-2.161768	4.099194	2.663844	50	Cl	2.011941	1.573620	2.494011

Table S5. Calculated bond lengths of [Ru(II)$Cl_2$$(DMSO)_2$(Hapbim)] (**1**)

Bond(Label-Label)		Length	Bond(Label-Label)		Length	Bond(Label-Label)		Length	Bond(Label-Label)		Length
1	2	1.3958	11	23	1.3736	22	28	2.0139	35	36	1.0993
1	6	1.4032	12	13	1.421	23	24	1.0217	35	37	1.101
1	7	1.095	12	14	1.4024	25	26	1.0436	35	38	1.1014
2	3	1.1404	12	22	1.405	25	27	1.0956	40	41	1.6953
2	8	1.0972	13	15	1.3633	25	28	2.0622	40	42	1.8532
3	4	1.4104	13	23	1.3789	28	29	2.4311	40	46	1.8542
3	11	1.4465	14	16	1.3979	28	30	2.2753	42	43	1.101
4	5	1.399	14	17	1.1053	28	40	2.2604	42	44	1.1004
4	25	1.429	15	18	1.3973	28	50	2.3827	42	45	1.1047
5	6	1.3992	15	19	10.95	30	31	2.8677	46	47	1.101
5	9	1.0977	16	18	1.4141	30	35	1.8581	46	48	1.0994
6	10	1.0954	16	20	1.0961	31	32	1.1007	46	49	1.1028
11	22	1.3593	18	21	1.0957	31	34	1.1033			

Table S6. Calculated bond angles of [Ru(II)$Cl_2$$(DMSO)_2$(Hapbim)] (**1**)

Angle	Angle(°)	Angle	Angle(°)	Angle	Angle(°)	Angle	Angle(°)	Angle	Angle(°)
2-1-6	119.6472	13-12-22	108.4024	13-23-24	125.7292	31-30-35	98.0305	40-42-43	105.7509
2-1-7	119.9513	14-12-22	131.6765	4-25-26	110.8371	31-30-39	106.8784	40-42-44	107.7763
6-1-7	120.3927	12-13-15	122.7304	4-25-27	116.1705	35-30-39	103.6146	40-42-45	104.9001
1-2-3	121.2688	12-13-23	105.7265	4-25-28	119.475	30-31-32	109.6523	43-42-44	113.5863
1-2-8	118.4966	15-13-23	131.5392	26-25-27	106.5838	30-31-39	106.718	43-42-45	111.1484
3-2-8	120.1726	12-14-16	119.507	26-25-28	101.1384	30-31-33	105.4984	44-42-45	112.9317
2-3-4	118.5738	12-14-17	122.2288	27-25-28	100.8673	30-31-34	112.1425	40-46-47	106.6368
4-3-11	119.3547	16-14-17	120-2418	22-28-25	89.9262	32-31-33	113.1287	40-46-48	107.2955
4-3-11	122.0685	13-15-18	116-7766	22-28-30	98.5914	32-31-34	111.2058	40-46-49	105.6739
3-4-5	120.0401	13-15-19	121.5655	22-28-40	92.3238	33-31-34	106.3393	47-46-48	113.0148
3-4-25	120.6805	18-15-19	121-3579	22-28-50	88.81116	30-35-36	106.4822	47-46-49	113.1157
5-4-25	119.2741	14-16-18	121-.6525	25-28-29	82.5553	30-35-37	106.3635	48-46-49	111.5938
4-5-6	120.8183	14-16-20	118.8662	25-28-40	84.9627	30-35-38	113.095		
4-5-9	118.9531	18-16-20	119.1499	25-28-50	90.9788	36-35-37	110.4091		
6-5-9	120.2274	15-18-16	121.0806	29-28-30	90.9254	36-35-38	112.7007		
1-6-5	119.6404	15-18-21	119.4934	29-28-40	88.8411	37-35-38	107.5394		
1-6-10	120.3974	16-18-21	119.4244	29-28-50	89.3543	25-40-41	116.0681		
5-6-10	119.9618	11-22-12	106.6651	30-28-40	94.8798	28-40-42	121.25354		
3-11-22	128.3836	11-22-28	122.1501	30-28-50	89.0056	28-40-46	107.1594		
3-11-23	121.6308	12-22-28	131.1489	28-30-31	116.1112	41-40-42	104.1181		
22-11-23	109.9795	11-23-13	109.2228	28-30-35	114.404	41-40-46	99.1016		
13-13-14	119.915	11-23-24	124.9127	28-30-39	115.7022	42-40-46	105.7509		

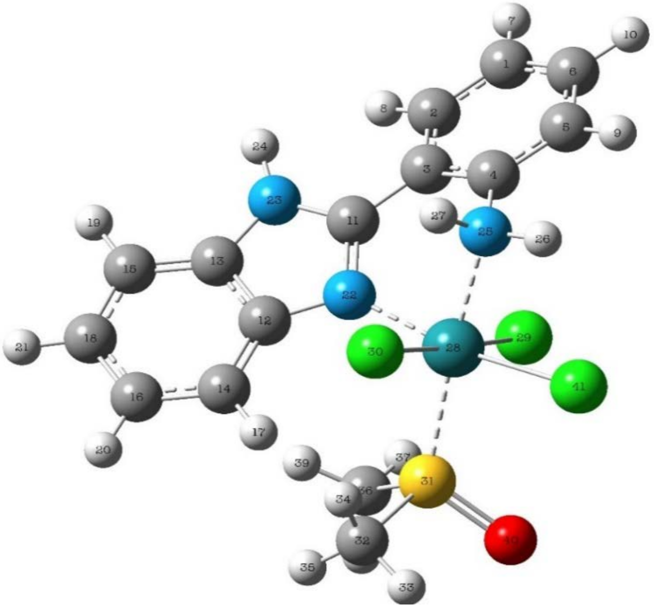

Fig S10. DFT calculated structure of [Ru(III)Cl_3(DMSO)(Hapbim)] (**2**)

Table S7. Calculated coordinates of [Ru(III)Cl_3(DMSO)(Hapbim)] (**2**)

		**Coordinates (Å)**					**Coordinates (Å)**		
**Atom Label**	**Element**	**X**	**Y**	**Z**	**Atom**	**Element**	**X**	**Y**	**Z**
1	C	4.253437	-1.12373	-0.494148	11	C	0.742178	0.040285	0.162242
2	C	2.893594	-1.13769	-0.172494	12	C	-1.29307	0.649601	0.798517
3	C	2.139836	0.048774	-0.201796	13	C	-1.35148	-0.7169	0.420686
4	C	2.770008	1.250527	-0.58528	14	C	-2.45774	1.327478	1.171672
5	C	4.128421	1.269778	-0.892712	15	C	-2.53809	-1.45089	0.446951
6	C	4.874551	0.08382	-0.845226	16	C	-3.65108	0.600691	1.198196
7	H	4.833912	-2.05098	-0.45421	17	H	-2.44626	2.401398	1.377898
8	H	2.427735	-2.07617	0.152779	18	C	-3.69008	-0.76937	0.852628
9	H	4.607575	2.214461	-1.178028	19	H	-2.56918	-2.50555	0.155576
10	H	5.943112	0.104706	-1.08425	20	H	-4.57939	1.10795	1.482338

Table S7 continued

		Coordinates (Å)					Coordinates (Å)		
**Atom Label**	**Element**	**X**	**Y**	**Z**	**Atom**	**Element**	**X**	**Y**	**Z**
21	H	-4.64584	-1.30319	0.887936	32	C	-1.88455	4.382271	2.701014
22	N	0.027681	1.085944	0.664315	33	H	-1.69749	5.37382	3.140173
23	N	-0.06447	-1.05305	0.034039	34	H	-2.05833	4.453866	1.614353
24	H	0.227357	-1.9533	-0.355348	35	H	-2.68942	3.852314	3.232749
25	N	1.993479	2.461333	-0.640633	36	C	-0.79159	1.984368	3.962513
26	H	2.594636	3.305857	-0.744257	37	H	0.146081	1.439015	4.155183
27	H	1.290054	2.462766	-1.410228	38	H	-1.24173	2.374732	4.8884
28	Ru	0.874158	2.875161	1.037939	39	H	-1.49714	1.376028	3.375311
29	Cl	2.430952	1.831484	2.476923	40	O	0.526845	4.391038	4.079985
30	Cl	-0.72623	3.771597	-0.460831	41	Cl	2.02908	4.943599	1.068811
31	S	-0.26957	3.466027	2.963619					

Table S8. Calculated bond lengths of [Ru(III)Cl_3(DMSO)(Hapbim)] (**2**)

Bond(Label-Label)		Length	Bond(Label-Label)		Length	Bond(Label-Label)		Length	Bond(Label-Label)		Length
1	2	1.3974	12	13	1.419	23	24	1.0233	32	35	1.1006
1	6	1.4026	12	14	1.3983	25	26	1.0418	36	37	1.1017
1	7	1.0947	12	22	1.3974	25	27	1.0426	36	38	1.101
2	3	1.406	13	15	1.3955	25	28	2.0595	36	39	1.1012
3	8	1.0971	13	23	1.3852	28	29	2.363			
3	4	1.4101	14	16	1.3975	28	30	2.3688			
3	11	1.4443	14	17	1.0936	28	31	2.3163			
4	5	1.3929	15	18	1.3986	28	41	2.3692			
4	25	1.4395	15	19	1.0946	31	32	1.8753			
5	6	1.4019	16	18	1.4135	31	36	1.8616			
5	9	1.097	16	20	1.0954	31	40	1.6541			
6	10	1.0952	18	21	1.0953	32	33	1.1005			
11	23	1.3647	22	28	2.0143	32	34	1.1028			

Table S9. Calculated bond angles of of [Ru(III)Cl_3(DMSO)(Hapbim)] (**2**)

Angle	Angle(°)	Angle	Angle(°)	Angle	Angle(°)
2-1-6	119.8114	16-14-17	121.0702	25-28-31	175.6843
2-1-7	119.9427	13-15-18	116.7132	25-28-41	85.4754
6-1-7	120.237	13-15-19	121.7294	29-28-30	176.0109
1-2-3	120.5615	18-15-19	121.555	29-28-31	86.084
1-2-8	119.3411	14-16-18	121.5337	29-28-41	93.2397
3-2-8	120.0276	14-16-20	119.1943	30-28-31	95.5019
2-3-4	119.0306	18-16-20	119.2682	30-28-41	90.4121
2-3-11	120.5697	15-18-16	121.3654	31-28-41	90.4102
4-3-11	120.3988	15-18-21	119.379	28-31-32	115.6862
3-4-5	120.5065	16-18-21	119.2479	28-31-36	112.4273
3-4-25	119.0935	11-22-12	106.9427	28-31-40	117.7738
5-4-25	120.3965	11-22-28	121.9877	32-31-36	102.8571
4-5-6	119.9911	12-22-28	131.0502	32-31-40	103.6459
4-5-9	119.6879	11-23-13	109.179	36-31-40	102.5889
6-5-9	120.3196	11-23-24	124.8871	31-32-33	103.7662
1-6-5	120.0731	13-23-24	125.7769	31-32-34	107.7832
1-6-10	120.2074	4-25-26	111.9874	31-32-35	109.0786
5-6-10	119.7195	4-25-27	113.1716	33-32-34	111.1935
3-11-22	126.5766	4-25-28	115.5215	33-32-35	111.4293
3-11-23	123.5732	26-25-27	108.3471	34-32-35	113.0833
22-11-23	109.8486	26-25-28	103.4026	31-36-37	104.4275
13-12-14	120.2409	27-25-28	103.5708	31-36-38	106.48
13-12-22	108.3024	22-28-25	84.188	31-36-39	109.4642
14-12-22	131.4127	22-28-29	89.8563	37-36-38	112.1111
12-13-15	122.4458	22-28-30	86.2659	37-36-39	111.4138
12-13-23	105.6507	22-28-31	99.979	38-36-39	112.4774
15-13-23	131.8838	22-28-41	169.3435		
12-14-16	117.6577	25-28-29	92.8383		
12-14-17	121.1747	25-28-30	85.845		

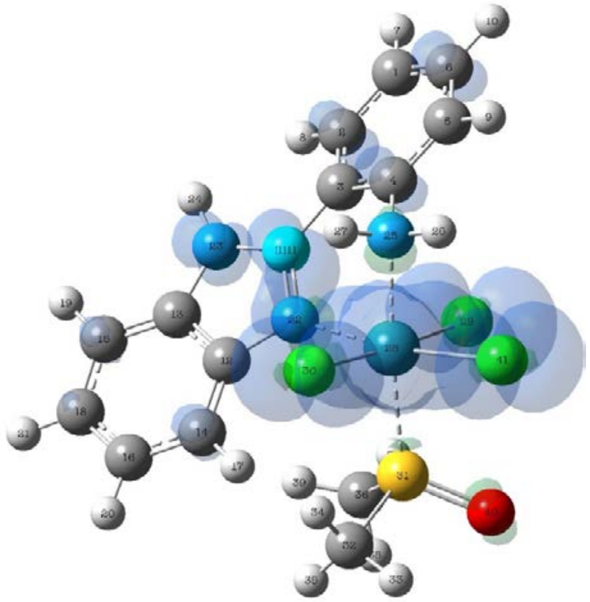

Fig S11. Calculated spin-density distribution of [Ru(III)Cl_3(DMSO)(Hapbim)] (**2**), shown with isovalue of 0.005

Table S10. Calculated Mulliken spin densities of [Ru(III)Cl_3(DMSO)(Hapbim)] (**2**)

Atom Label	Atom	Mulliken Spin Densities	Atom Label	Atom	Mulliken Spin Densities	Atom Label	Atom	Mulliken Spin Densities	Atom Label	Atom	Mulliken Spin Densities
1	C	-0.0029	12	C	-0.0014	23	N	0.0122	34	H	0.0000
2	C	0.0082	13	C	0.0004	24	H	-0.0003	35	H	-0.0001
3	C	-0.0023	14	C	0.0059	25	N	-0.0074	36	C	-0.0013
4	C	0.0080	15	C	0.0057	26	H	-0.0003	37	H	0.0000
5	C	-0.0032	16	C	0.0028	27	H	-0.0001	38	H	-0.0002
6	C	0.0107	17	H	-0.0004	28	Ru	0.7401	39	H	0.0001
7	H	0.0001	18	C	0.0045	29	Cl	0.0799	40	O	-0.0044
8	H	-0.0004	19	H	-0.0003	30	Cl	0.0597	41	Cl	0.0730
9	H	0.0001	20	H	-0.0002	31	S	-0.0077			
10	H	-0.0005	21	H	-0.0003	32	C	-0.0022			
11	C	0.0267	22	N	-0.0020	33	H	-0.0001			
